# Optical Detection of Cellular Signals at Material Interfaces

**DOI:** 10.1002/adhm.71670

**Published:** 2026-09-06

**Authors:** Xuchen Ren, Jitong Ren, Zixin Zhang, Koney Eungkon Kim, Francis L. Hong, Yuecheng Peter Zhou

**Affiliations:** ^1^ Department of Materials Science and Engineering The Grainger College of Engineering University of Illinois Urbana‐Champaign Urbana Illinois USA; ^2^ Department of Chemical and Biomolecular Engineering College of Liberal Arts & Sciences University of Illinois Urbana‐Champaign Urbana Illinois USA; ^3^ Department of Bioengineering The Grainger College of Engineering University of Illinois Urbana‐Champaign Urbana Illinois USA; ^4^ Materials Research Laboratory University of Illinois Urbana‐Champaign Urbana Illinois USA; ^5^ Beckman Institute for Advanced Science and Technology University of Illinois Urbana‐Champaign Urbana Illinois USA

**Keywords:** biochemical markers, bioelectronics, biointerface, biophotonics, cell signaling, label free biosensing, materials science, nanotechnology, optical sensing

## Abstract

Cellular functions are dynamically regulated by electrical, chemical, and mechanical signals. Optical methods provide high spatial resolution and flexibility for detecting and understanding cellular signals, but most existing approaches rely on fluorescent reporters, which are often limited by photobleaching, phototoxicity, and potential perturbation of native cellular behaviors. In recent years, materials‐based optical detection has emerged as a promising strategy, which transduces subtle cellular signals into measurable optical readouts without introducing fluorescent labels or the need for genetic modification. However, this field is still at a nascent stage, as many materials platforms are constrained by limited detection sensitivity, specificity, spatiotemporal resolution, and compatibility with different biological systems. Here, we discuss recent advances in optical methods for detecting cellular signals harnessing cell‐material interfaces. In particular, we connect the structure‐property relationships of polymers, liquid crystals, carbon‐based materials, two‐dimensional materials, quantum materials, and plasmonic metals to their optical detection mechanisms. We then summarize the applications of these materials in optical detection of bioelectric, biomechanical, and biochemical signals. We anticipate that this review will bridge materials science with cell biology, and guide the rational design of next‐generation material‐based optical detection platforms for minimally invasive, high‐sensitivity interrogation of cellular signals.

## Graphical abstract

Emerging functional materials are transforming optical detection of cellular signals. This review highlights optical techniques that exploit the unique optical properties of diverse materials to detect and quantify cellular electrical, chemical, and mechanical signals, and discusses key opportunities and challenges. Bridging materials science and biology, this review aims to inspire advances in materials design and optical instrumentation for biomedical applications.

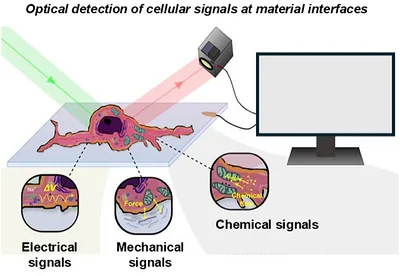

**Abbreviations**
3D‐SIM‐TFM: 3D structured illumination microscopy with traction force microscopy
4‐ATP: 4‐Aminothiophenol
4‐NTP: 4‐Nitrothiophenol
ACSF: Artificial cerebrospinal fluid
ATP: Adenosine triphosphate
AuNP: Gold(Au) nanoparticle
AuNR: Gold(Au) nanorod
Aβ: Amyloid beta
BF: Bright field
BSA: Bovine serum albumin
CAGE: Critically‐coupled waveguide‐amplified graphene electric field
CCCP: Carbonyl cyanide *m*‐chlorophenyl hydrazone
CF‐SPN: CRET‐FRET semiconducting polymer nanoparticle
CFU: Colony‐forming unit
ClO^−^
: Hypochlorite
CMOS: Complementary metal‐oxide‐semiconductor
CoPhMoRe: Corona phase molecular recognition
CPPO: bis‐(2,4,5‐Trichloro‐6‐(pentyloxycarbonyl)phenyl)oxalate
CRET: Chemiluminescence resonance energy transfer
cTFM: Confocal traction force microscopy
Cy5: Cyanine‐5
dcDNA‐Ti_3_C_2_
: Chimeric DNA‐functionalized MXene probe
DHF: Deformed‐helix ferroelectric
DIC: Differential interference contrast
DMAB: *p*,*p*′‐Dimercaptoazobenzene
DNA: Dioxyribonucleic acid
ECM: Extracellular matrix
ECORE: Electrochromic optical recording
EGFP: Enhanced green fluorescent protein
EM 1^2+^
: Dicationic 1,1,4,4‐tetraarylbutadiene
EM F1^2+^
: Dicationic 1,1,4,4‐tetra(pentafluorophenyl)butadiene
EM F2: 1,1,4,4‐Tetraarylbutadiene
EOT: Extraordinary optical transmission
ERISM: Elastic resonator interference stress microscopyethylidene)‐1‐cyclohexen1yl]‐1,3,3‐trimethyl‐3H‐indolium chloride
FAM: 6‐Carboxyfluorescein
FBSR TFM: Fluctuation‐based super‐resolution traction force microscopy
FCCP: Carbonyl cyanide *p*‐trifluoromethoxyphenylhydrazone
FLC: Ferroelectric liquid crystal
FOV: Field of view
fps: Frames per second
FRET: Förster resonance energy transfer
FSCV: Fast‐scan cyclic voltammetry
GO: Graphene oxide
GRIN: Gradient refractive index
GT: Guanine‐thymine
GTP: Guanosine triphosphate
H_2_O_2_
: Hydrogen peroxide
H_2_S: Hydrogen sulfide
HEK 293: Human embryonic kidney 293 cells
HEPG2: Human hepatocellular carcinoma cell Line
hiPSC: Human induced pluripotent stem cell
IR775COOH: 2‐[2‐[2‐(β‐Carboxyethylthio)‐3‐(2‐(1,3‐dihydro‐1,3,3‐trimethyl‐2H‐indol‐2‐ylidene)
ITO: Indium tin oxide
LC: Liquid crystal
LSPR: Localized surface plasmon resonance
MCF‐7: Michigan Cancer Foundation‐7 breast cancer cell line
MEH‐PPV: Poly[2‐methoxy‐5‐(2‐ethylhexyloxy)‐1,4‐phenylenevinylene]
miR‐21: MicroRNA‐21
MoS_2_
: Molybdenum disulfide
MPBN: 4‐Mercaptobenzonitrile
MUC1: Mucin 1
NanoDRONE: Dual‐color SPN‐based NIR nanoprobe
NIR: Near‐infrared
NIR775: Silicon 2,3‐naphthalocyanine bis(trihexylsilyloxide)
nIRCats: Near‐infrared catecholamine nanosensors
NP: nanoparticle
NV: Nitrogen vacancy
OCEANs: Organic electro‐scattering antennas
ODMR: Optically detected magnetic resonance
ONOO^−^
: peroxynitrite
ox‐PPE: Oxidized poly(3,4‐propylenedioxythiophene‐alt‐3,4‐ethylenedioxythiophene)
P(OE3)‐E: Copolymer of oligoether‐functionalized 3,4‐propylenedioxythiophene with unsubstituted 3,4‐ethylenedioxythiophene
PAAm: Polyacrylamide
PANI: Polyaniline
PBS: Phosphate‐buffered saline
PCFDP: 9,9′‐Dihexyl‐2,7‐bis(1‐cyanovinylene)fluorenylene‐alt‐co‐2,5‐bis(*N*,*N*′‐diphenylamino)‐1,4‐phenylene
PDMS: Polydimethylsiloxane
PEDOT: Poly(3,4‐ethylenedioxythiophene)
PEG: Polyethylene glycol
P‐EIM: Plasmonic electrochemical impedance microscopy
PFODBT: Poly(2,7‐(9,9‐dioctylfluorene)‐alt‐4,7‐bis(thiophen‐2‐yl)benzo‐2,1,3‐thiadiazole)
PL: Photoluminescence
PPE: Poly(3,4‐propylenedioxythiophene‐alt‐3,4‐ethylenedioxythiophene)
PSM: Plasmonic scattering microscopy
PSS: Polystyrene sulfonate
QD: Quantum dot
RNA: Ribonucleic acid
RNS: Reactive nitrogen species
ROS: Reactive oxygen species
SBR: Signal‐to‐background ratio
SERS: Surface‐enhanced Raman scattering/spectroscopy
SmA: Smectic A
SmC: Smectic C
SmC*: Chiral Smectic C
SNP: Sodium nitroprusside
SNR: Signal‐to‐noise ratio
SPE: Screen‐printed electrodes
SPHM: Surface plasmon holographic microscopy
SPN: Semiconducting polymer nanoparticle
SPORT: Single‐particle orientation and rotational tracking
SPR: Surface plasmon resonance
SPRi: SPR imaging
SPRIE: SPR imaging ellipsometry
STED: Stimulated emission depletion
STFM: Super‐resolved traction force microscopy
SWCNT: Single‐walled carbon nanotube
TFM: Traction force microscopy
TGF‐β: Transforming growth factor‐β
TIRF: Total internal reflection fluorescence microscopy
TNF‐α: Tumor necrosis factor alpha
T_NV_
: Photon count‐derived temperature estimate
TTX: Tetrodotoxin
UV–Vis‐NIR: Ultraviolet–visible‐near‐infrared
WARP: Wavelength‐alternating resonant pressure microscopy

## 1 Introduction

Cells sense and respond to diverse electrical, mechanical, and chemical signals that govern their functions and intercellular communication [1]. Bioelectric signals such as action potentials enable rapid information transfer in electrogenic cells such as neurons and cardiomyocytes [2, 3]. Malignant cells such as cancer cells can also exhibit altered electrical properties and form abnormal electrochemical coupling with healthy cells [4]. Mechanical cues such as forces arising from cell adhesion and matrix stiffness regulate cytoskeletal integrity and drive cellular processes including differentiation, growth, and migration [5]. Chemical signals such as neurotransmitters, hormones, and reactive oxygen and nitrogen species mediate neuronal communication, metabolic regulation, and immune responses [6, 7, 8, 9]. Together, bioelectric, biomechanical, and biochemical signals coordinate complex behaviors across cells and tissues [10, 11, 12]. A comprehensive understanding of these signals is crucial for elucidating disease progression, developing early diagnostics, and guiding drug discoveries [13, 14, 15].

Despite their importance, detecting and understanding cell signals remains challenging. These signals often exhibit small amplitudes, occur on millisecond timescales, and display heterogeneous spatial distributions within cells and tissues. Further, many interactions are transient that can be easily perturbed during measurements, making them difficult to be captured without altering the native cellular behavior. Among the existing detection methods, optical techniques possess unique advantages. Optical detection methods offer excellent spatiotemporal resolution for direct visualization of cellular processes in real time [16]. The inherent spatial flexibility of light also supports a broad range of in vitro and in vivo conditions [17, 18]. In addition, probing cellular signals using light minimizes invasiveness and helps preserve native cellular behavior during measurement [19].

However, most current optical methods for detecting cell signals heavily rely on fluorescent reporters such as proteins or small‐molecule dyes [20]. For example, calcium‐ [21, 22], dopamine‐ [23], glutamate‐ [24], or voltage‐sensitive domains [25, 26] can be fused to green fluorescent proteins to create recombinant reporters that visualize intracellular calcium dynamics [21, 22], neurotransmitter activities [23, 24], and transmembrane potential changes [27]. Fluorescent proteins can also be configured for Förster resonance energy transfer (FRET) to monitor intracellular tension and other biomechanical cues [28, 29]. These reporters can be genetically encoded for cell‐type‐specific imaging in vitro and in vivo, but can be susceptible to photobleaching and phototoxicity [30, 31], especially when expressed within the cytosol or inserted into the cell membrane. Small‐molecule dyes enable simpler sample preparation and faster response time than protein‐based reporters [32, 33], but similar limitations make them challenging to be used for long‐term measurements [34].

Recent advances in materials science enable rational design of materials with tailored optical properties. By engineering the cell‐material interface, materials spanning from soft polymers to hard metals can be coupled to live cells and tissues [35, 36, 37, 38]. Bioelectric, biomechanical or biochemical signals can be transduced into robust optical readouts without inserting fluorescent proteins or small‐molecule dyes into the cells [39, 40]. This approach expands the capabilities of optical detection toolkits to unlock new opportunities for high‐resolution, minimally invasive monitoring of cellular signals. In addition, advances in materials development are driving a growing need for optical detection platforms that can better harness the unique optical properties of different materials [41, 42].

In this article, we review emerging optical detection techniques that exploit the unique optical properties of different materials to detect and quantify cellular electrical, chemical, and mechanical signals. Our goal is to bridge materials science and biology, and inspire new directions in materials design, optical instrumentation, and their biomedical applications (Figure 1). We organize the review by classes of materials beginning with organic materials, including conjugated polymers, polymer elastomers, and liquid crystals. We then discuss carbon‐based materials such as graphene and carbon nanotubes, followed by inorganic two‐dimensional (2D) materials (e.g., molybdenum disulfide, MXene) and quantum materials (e.g., diamonds with nitrogen‐vacancy centers). Finally, we discuss metals employed in surface plasmon resonance, optical interference structures, and Raman scattering. Together, we highlight the versatility and transformative potential of materials‐based optical approaches in understanding cellular signals.

**FIGURE 1:**
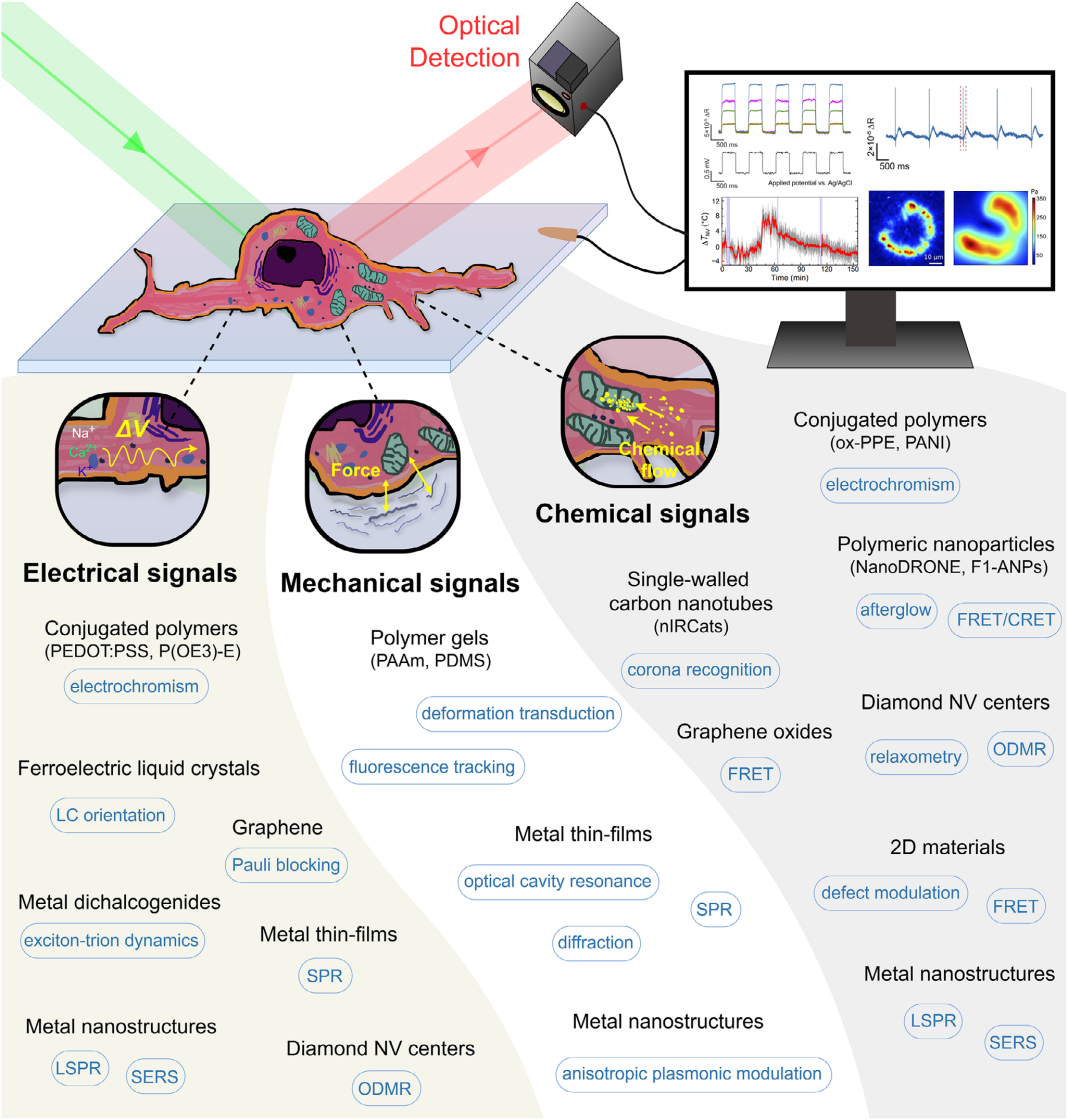
Overview of topics covered in this review. Optical detection of electrical, mechanical, and chemical signals in cells and tissues at material interfaces. Schematic created using draw.io. Top‐right inset adapted from Ref. [63] under the CC BY‐NC 4.0 license. Copyright 2025, The Author(s); adapted from Ref. [211] under the CC BY 4.0 license. Copyright 2020, The American Association for the Advancement of Science; and adapted from Ref. [126] under the CC BY 4.0 license. Copyright 2020, American Chemical Society.

## 2 Polymers

Polymers are macromolecular materials formed by covalently linking repeating monomer units into long chains. Their emergent properties are dictated by molecular weight, chain architecture, and structural arrangement [43, 44]. We classify polymers into conjugated and non‐conjugated polymers based on their backbone electronic structures. Most conjugated polymers feature delocalized π‐electron systems arising from alternating single and double bonds along the backbone [45], which leads to semiconducting behavior and strong light‐material interactions [46]. This enables direct transduction of bioelectric and biochemical signals into optical readouts via redox‐coupled or photoexciton processes. In contrast, non‐conjugated polymers possess σ‐bonded backbones with localized electronic states and are typically electrically insulating. They are predominantly employed as hydrogels [47] or elastomers [48] that provide optically transparent, mechanically tunable, and biocompatible matrices for regulating biomechanical signal transduction.

### 2.1 Conjugated Polymers

𝜋‐Conjugated polymers such as polythiophene and polyaniline possess extended π‐electron delocalization along their backbone. The structure and function of conjugated polymers can be precisely controlled through backbone and side chain design [49, 50, 51]. They are also mechanically flexible and solution‐processable, which makes them well‐suited for integration with cells and tissues [52].

Conjugated polymers strongly interact with light. Many of them possess large extinction coefficients and high photoluminescence (PL) quantum yields [46, 53]. They also exhibit highly efficient energy transfer behavior, in which excitation energy can migrate along the polymer chain and be transferred to nearby molecules [54]. This process is closely related to Förster resonance energy transfer (FRET) [55] and can enable sensitive detection of short‐range (1–10 nm) interactions between biomolecules [56]. In addition, the optical absorption of conjugated polymers is strongly coupled to their redox states, giving rise to their electrochromic behavior [57]. Doping generates charge carriers, typically polarons and bipolarons, along the conjugated backbone. This increases electrical conductivity while altering the optical absorption spectrum. Consequently, an applied voltage can reversibly modulate the optical absorption and color of conjugated polymers.

#### 2.1.1 Bioelectric Signal Detection

Poly(3,4‐ethylenedioxythiophene) (PEDOT), a derivative of polythiophene, when doped with polyelectrolyte polystyrene sulfonate (PSS), forms PEDOT:PSS (Figure 2a). PEDOT:PSS is well known for its electrical conductivity, which can exceed 1000 S/cm following post‐treatment with solvents such as ethylene glycol or dimethyl sulfoxide [58, 59, 60]. PEDOT:PSS is also electrochromic. It exhibits drastic color changes under different applied voltages. It appears blue when an oxidizing voltage is applied and purple when a reductive voltage is applied (Figure 2b). Indeed, when external voltages from −700 mV to +600 mV were applied to a PEDOT:PSS thin film, its ultraviolet–visible‐near‐infrared (UV–vis‐NIR) absorption spectrum shifts significantly (Figure 2c).

**FIGURE 2:**
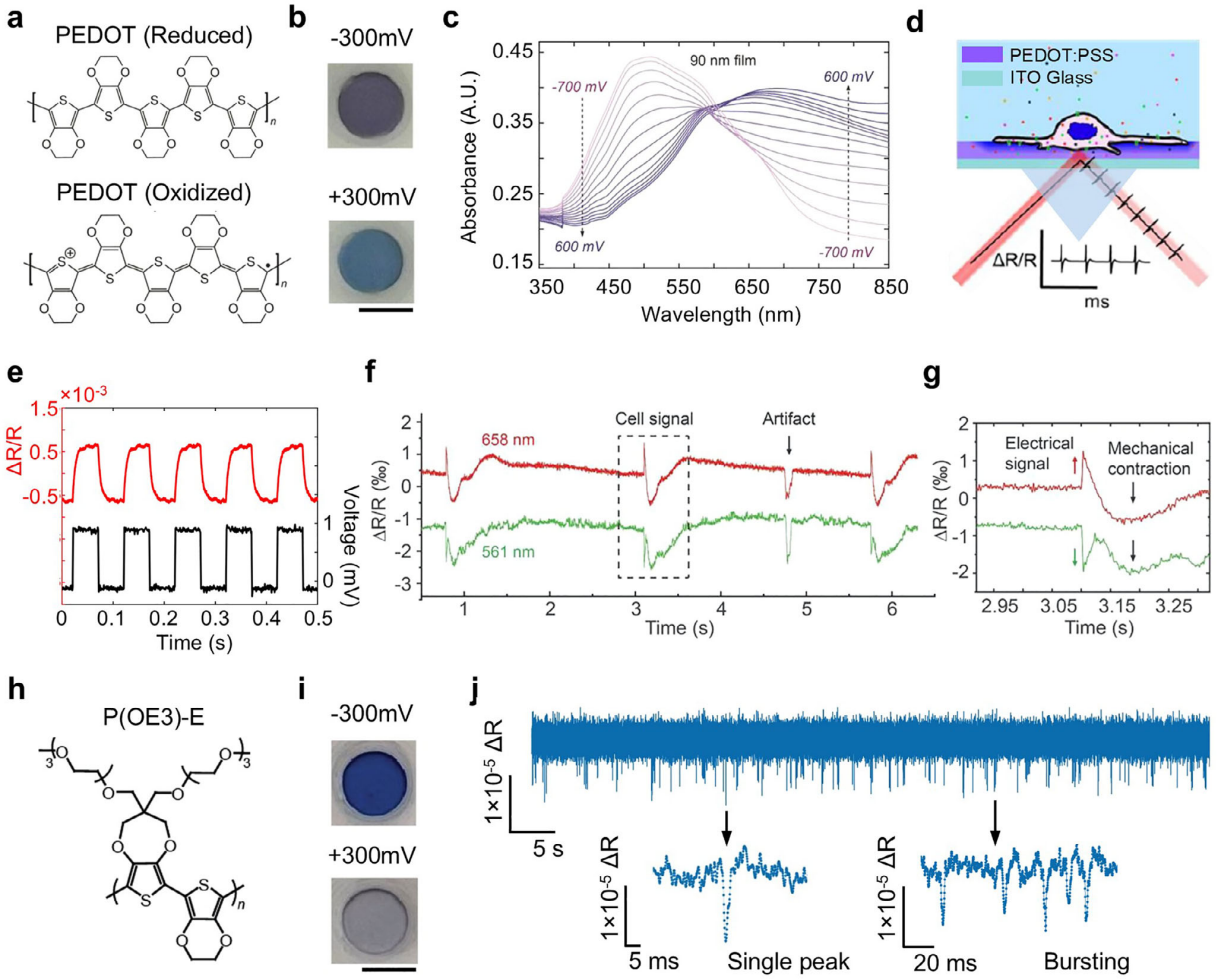
Detecting bioelectric signals using electrochromic polymer films. (a) The chemical structure of PEDOT:PSS. (b) Color transitions of a PEDOT:PSS film at +300 mV (oxidized state, blue) and −300 mV (reduced state, purple). Scale bar = 5 mm. (c) UV–visible‐near infrared absorption spectrum of PEDOT:PSS thin film when an external voltage from +600 to −700 mV versus an Ag/AgCl reference electrode was applied in HEPES‐buffered Tyrode's solution. (d) Schematic representation of ECORE. (e) ECORE recording signal in response to applied 1 mV, 1 Hz squarewave. (f) Dual‐color ECORE recording traces using 561 nm (green) and 658 nm (red) lasers. The recorded electrical signal (sharp spikes) shows opposite polarity. The recorded mechanical contraction (slower baseline shift) and artifacts show the same polarity. (g) Zoomed‐in view of the dashed box in (f). (h) The chemical structure of P(OE3)‐E. (i) Color transitions of a P(OE3)‐E film at +300 mV (oxidized state, transparent) and −300 mV (reduced state, blue) Scale bar = 5 mm. (j) ECORE recording trace of a cultured rat hippocampal neuron that shows burst firing. Panels (a, c, f, g) reproduced from Ref. [61] under the CC BY‐NC‐ND 4.0 license. Copyright 2022, American Chemical Society. Panels (d and e) adapted from Ref. [62]. Panels (b, h–j) adapted from Ref. [63] under the CC BY‐NC 4.0 license. Copyright 2025, The Author(s).

Leveraging the electrochromic properties of PEDOT:PSS, Alfonso and coworkers demonstrated a label‐free optical recording approach for bioelectric signals named ElectroChromic Optical Recording (ECORE) [62]. Bioelectric signals such as cell extracellular action potentials are typically on the order of tens to hundreds of microvolts and cannot induce a visible color change of the polymer film. Therefore, a sensitive optical setup based on a prism‐coupled total internal reflectance configuration was built to detect such small bioelectric signal‐induced color changes. In this work, cells were cultured on PEDOT:PSS thin films electropolymerized on indium tin oxide (ITO)‐coated glass substrates. A 658 nm probing laser beam was focused to a ∼25 µm spot that precisely probes the cell‐polymer interface. The reflected beam from the sample was then collected by a differential photodetector for laser noise rejection and allowed sub‐millisecond temporal resolution (Figure 2d). When a 1 mV, 1 Hz squarewave potential was applied to the film in a cell‐free environment, ECORE detected a fractional reflectance change, Δ*R*/*R*, of ∼3.0 × 10^−3^ with a root‐mean‐square noise of less than 2.0 × 10^−5^, which corresponds to a voltage recording sensitivity of ∼6.7 µV (Figure 2e).

Zhou et al. further developed a dual‐color ECORE recording platform leveraging the opposite optical responses of PEDOT:PSS thin films at 561 and 658 nm under the same applied voltage (Figure 2c). This approach increased the effective voltage detection sensitivity to ∼5 µV [61]. Figure 2f shows two time‐synchronized recording traces from a human‐induced pluripotent stem cell (hiPSC)‐derived cardiomyocytes cultured on PEDOT:PSS thin films, recorded using 561 and 658 nm lasers. Here, bioelectric signals show *opposite* optical responses, while recording noise or artifacts show the *same* optical response. Notably, both the electrical activity and mechanical contraction of cardiomyocytes were detected, and can be distinguished and decoupled under dual‐color ECORE (Figure 2g). This is because bioelectric signals change the optical absorbance of the electrochromic thin film, leading to opposite responses in the two recording channels, whereas mechanical contractions alter the refractive index at the cell–polymer interface, leading to similar responses in both channels. Moreover, bioelectric signals and mechanical contractions show different timescales. Bioelectric signals appear as sharp spikes on the order of a few milliseconds and precede the slow mechanical contraction signals on the order of hundreds of milliseconds due to cardiac excitation–contraction coupling [64]. Recent work by Ren and colleagues improved the detection sensitivity of ECORE to ∼3 µV by tuning the electropolymerization of PEDOT:PSS [65].

In addition to PEDOT:PSS, propylenedioxythiophene (ProDOT)‐based conjugated polymers also exhibit strong electrochromic color contrast [66]. Zhou and colleagues used a copolymer of oligoether‐functionalized 3,4‐propylenedioxythiophene with unsubstituted 3,4‐ethylenedioxythiophene [P(OE3)‐E] (Figure 2h), which transitions from dark blue in the reduced state to transparent in the oxidized state, for ECORE (Figure 2i). Using P(OE3)‐E, the authors achieved a detection sensitivity of ∼3 µV and recorded spontaneous extracellular action potentials from rat hippocampal neurons 21 days after seeding on the polymer films (Figure 2j) [63]. The sub‐millisecond (0.1 ms) temporal resolution of ECORE and the fast redox switching of P(OE3)‐E films allowed the detection of neuronal burst firing as fast as 93 Hz (Figure 2j, inset). Beyond cultured cells, ECORE can also interface with tissues, such as cultured embryonic rat hearts and postnatal rat brain slices, to record their field potentials [62, 63]. Overall, ECORE enables label‐free optical recording of bioelectric signals with high temporal resolution. Since the laser beams can be placed on arbitrary cells in the culture or tissue, ECORE also allows excellent recording spatial flexibility. Moreover, the biocompatibility of 𝜋‐conjugated electrochromic polymers enables long‐term recording of cardiac and neuronal electrical signals over months of time for the same device without significant degradation in recording signal‐to‐noise ratio (SNR) [61].

#### 2.1.2 Biochemical Signal Detection

##### 2.1.2.1 Bacterial Metabolite

Conjugated electrochromic polymers can also convert bacterial metabolic activities into optical readouts. Wu et al. advanced this concept by introducing a water‐soluble electrochromic polymer poly(3,4‐propylenedioxythiophene‐alt‐3,4‐ethylenedioxythiophene) (PPE), which can be oxidized by ambient oxygen into its bipolaronic state (ox‐PPE) [67]. PPE exhibited excellent stability in aqueous solution and possessed neutral, polaron, and bipolaron states (Figure 3a) with distinct colors as indicated by the spectroelectrochemistry (Figure 3b). In the reduced state, PPE exhibited a strong absorption band around 600 nm, partial oxidation generated polaron bands near 1000 nm, and full oxidation produced a broad bipolaron band in the near‐infrared (NIR) region, which shows its sensitivity to electron transfer in aqueous media. When introduced into *Escherichia coli* suspensions, ox‐PPE directly reported bacterial metabolism through measurable color changes, producing spectroscopic responses within 30 min and enabling detection down to 10^4^ cells mL^−1^. Increasing glucose concentration accelerated bacterial metabolic activity and induced progressively stronger reduction of ox‐PPE, which was observed both spectroscopically and visually (Figure 3c). In addition, ox‐PPE allows distinguishing between different bacterial species. For instance, Gram‐negative strains secrete higher levels of glutathione than Gram‐positive strains, therefore producing a stronger color change through more pronounced reduction of ox‐PPE.

**FIGURE 3:**
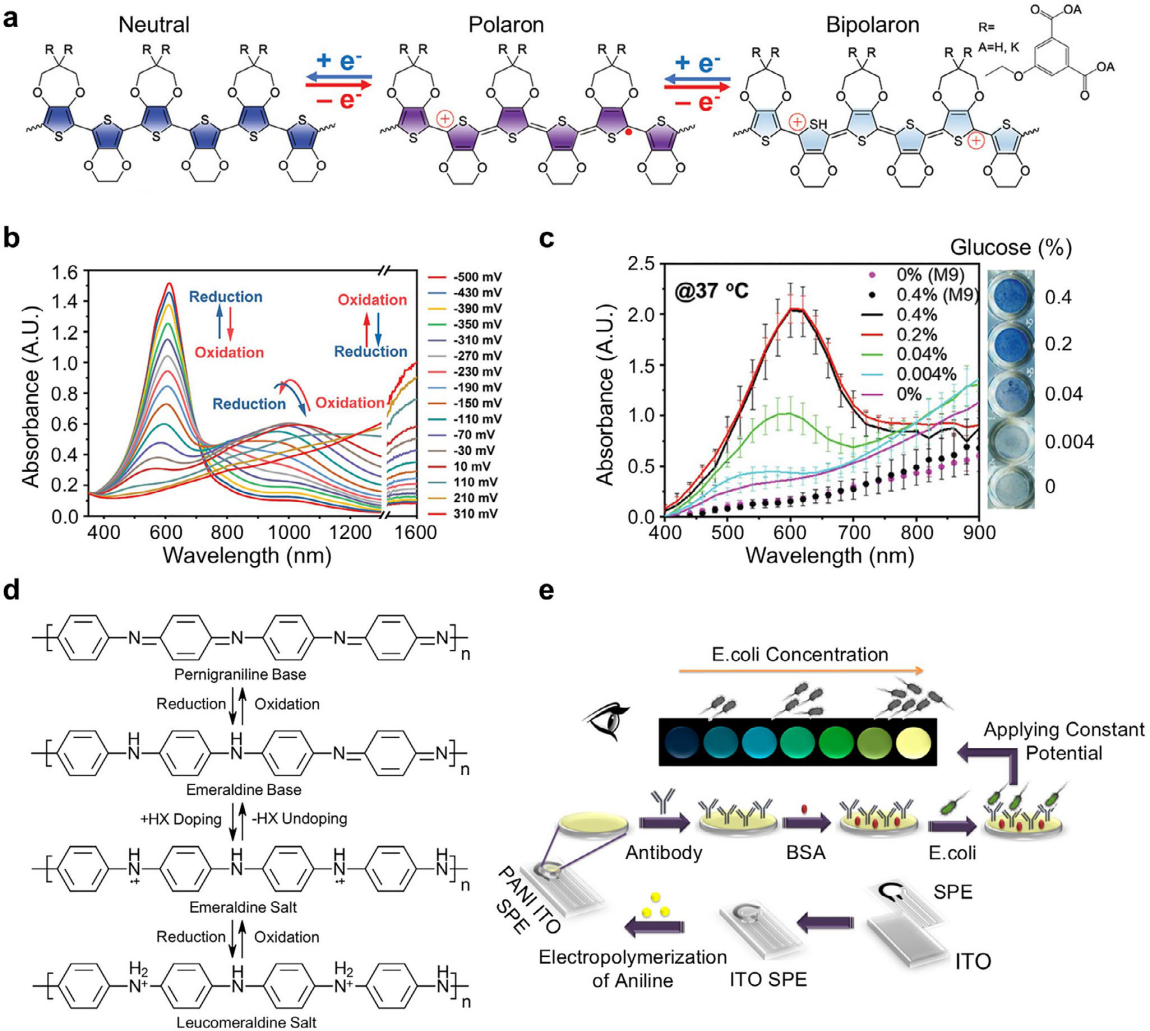
Detecting bacterial metabolites using electrochromic polymer films. (a) Schematic of PPE in neutral, polaron, and bipolaron states. (b) Spectroelectrochemistry of PPE showing voltage‐dependent absorption changes. (c) Absorption spectra of ox‑PPE after 2 h incubation with *Escherichia coli* (10^8^ cells/mL) in medium containing varying glucose concentrations (0%–0.4%) at 37°C. (d) Different PANI electrochemical states. (e) Schematic of electrochromic sensor fabrication. The sensor changes color in response to *E. coli* concentrations under a constant potential that enables visual readout of bacterial levels through the color shift of the PANI film. Panels (a–c) adapted with permission from Ref. [67]. Copyright 2020, Wiley‐VCH. Panels (d and e) adapted with permission from Ref. [68]. Copyright 2019, American Chemical Society.

Ranjbar et al. fabricated an immunosensor chip using polyaniline (PANI) thin films [68]. The electrochromic behavior of PANI arises from its multiple redox states, including leucoemeraldine salt (yellow), emeraldine salt (green/blue), emeraldine base (blue), and pernigraniline base (blue‐violet), each associated with distinct electronic structures (Figure 3d). Building on this property, the sensor was fabricated by electropolymerizing aniline on ITO screen‐printed electrodes (SPEs) to form PANI films, followed by modification with bovine serum albumin (BSA) and anti‐*E. coli* antibodies (Figure 3e). The immobilized antibodies enabled selective binding of *E. coli* cells on the PANI surface, which increased interfacial resistance and hindered the electrochromic transition of the polymer under an applied potential. As a result, the characteristic yellow‐to‐blue color change of PANI was progressively suppressed with increasing bacterial concentration. This provided a sensitive and selective way to detect *E. coli* in water samples through visible color changes, with a detection limit of 100 CFU (colony‐forming unit) mL^−1^ by naked‐eye inspection and 10 CFU mL^−1^ by image analysis. By linking biochemical activity to electrochromic changes, conjugated polymers can transduce bacterial metabolism into optical signals for rapid, label‐free bacterial detection and antibiotic susceptibility testing [67, 68, 69]. These optical signals can be captured using a variety of detectors, including cameras, smartphones, or direct visual inspection [70].

##### 2.1.2.2 Reactive Oxygen and Nitrogen Species (RONS)

Reactive oxygen and nitrogen species (RONS) including hydrogen peroxide (H_2_O_2_), hypochlorite (ClO^−^), and peroxynitrite (ONOO^−^) are oxidative mediators that regulate physiological signaling networks [71]. When produced in excess, RONS can disrupt redox homeostasis and contribute to chronic inflammation, cancer progression, neurodegeneration, and cardiovascular disease [72, 73]. Pu et al. introduced NanoDRONE, a fluorescent dye‐doped conjugated polymer nanoparticle (NP) probe to detect RONS [74]. The design integrates a photostable conjugated polymer 9,9′‐dihexyl‐2,7‐bis(1‐cyanovinylene)fluorenylene‐alt‐co‐2,5‐bis(*N*,*N*′‐diphenylamino)‐1,4‐phenylene (PCFDP) core with pegylated phospholipids to yield NPs that passively accumulate in inflamed tissues. Surface conjugation with a cyanine dye 2‐[2‐[2‐(β‐carboxyethylthio)‐3‐(2‐(1,3‐dihydro‐1,3,3‐trimethyl‐2H‐indol‐2‐ylidene)ethylidene)‐1‐cyclohexen1yl]‐1,3,3‐trimethyl‐3H‐indolium chloride (IR775COOH) creates a FRET relay in which fluorescence at ∼818 nm dominates in the RONS‐free state (Figure 4a). Upon exposure to RONS, the cyanine dye is selectively oxidized and is fragmented, eliminating FRET and thereby enhancing polymer emission at ∼678 nm. Such spectral switch enables nanomolar sensitivity to RONS and can be selectively activated in lipopolysaccharide‐induced peritonitis mouse models with inflamed macrophages (Figure 4b). These results establish conjugated polymer NPs as robust probes for RONS imaging, while mitigating fluorescence loss caused by oxidative quenching and degradation [75]. While NanoDRONE demonstrates such advantages, the cyanine dye reporter can undergo oxidative cleavage by multiple highly reactive oxidants. This limits its chemical specificity for identifying different oxidant species.

**FIGURE 4:**
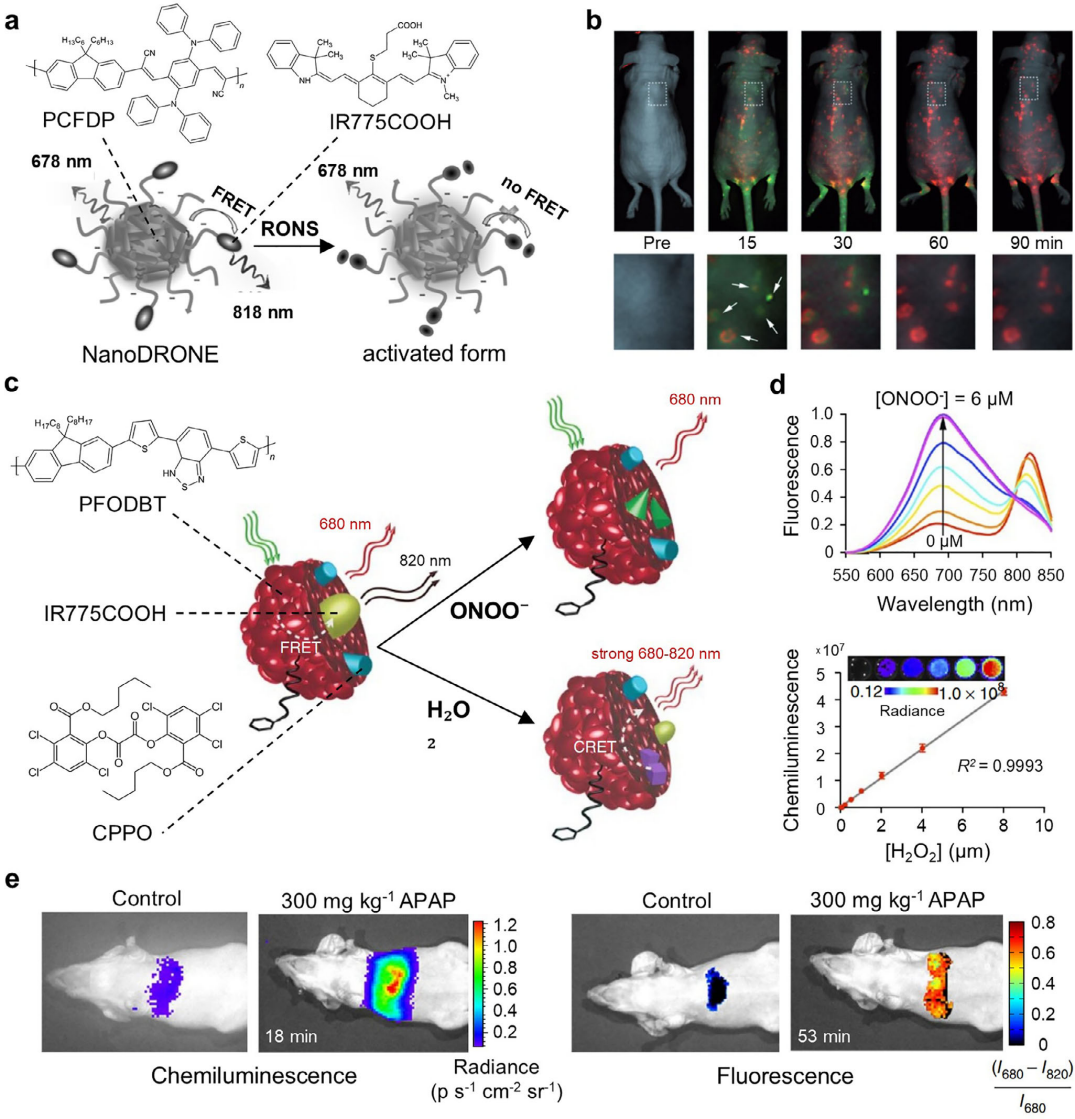
Detecting reactive oxygen and nitrogen species (RONS) using conjugated polymer nanoparticles. (a) Assembly and working mechanism of the NanoDRONE ratiometric RONS probe. PCFDP semiconducting polymer nanoparticles are conjugated with IR775COOH to form a FRET relay that produces ∼818 nm emission, which is disrupted in the presence of RONS. (b) In vivo detection of RONS with NanoDRONE following systemic *Corynebacterium bovis* infection. Overlaid images display unactivated probe (green) and RONS‐activated probe (red). Dashed boxes are zoomed‐in below each image with arrows indicating localized regions of bacterial infection. (c) Dual‐sensing modes of the CF‐SPN nanosensor, composed of PFODBT, IR775COOH, and CPPO within PEG, undergoing ONOO^−^‐induced FRET quenching and H_2_O_2_‐induced CRET. (d) Fluorescence spectra of CF‐SPN under increasing ONOO^−^ concentrations (top) and H_2_O_2_‐dependent chemiluminescence intensity of CF‐SPN (bottom). (e) In vivo imaging of APAP‐induced oxidative stress using CF‐SPN nanosensors. Chemiluminescence (left) and fluorescence (right) images are collected after 18 and 53 min after CF‐SPN injection, respectively. Panels (a and b) adapted with permission from Ref. [74]. Copyright 2013, WILEY‐VCH. Panels (c–e) reproduced with permission from Ref. [76]. Copyright 2014, Springer Nature.

To selectively detect H_2_O_2_ and ONOO^−^ molecules, Shuhendler et al. further developed a dual‐channel nanosensor (CF‐SPN) that combines FRET with chemoluminescence resonance energy transfer (CRET) [76]. CF‐SPN consists of a conjugated polymer core poly(2,7‐(9,9‐dioctylfluorene)‐alt‐4,7‐bis(thiophen‐2‐yl)benzo‐2,1,3‐thiadiazole) (PFODBT) that serves as FRET energy donor and CRET energy acceptor, a galactose‐functionalized polyethyleneglycol (PEG) group to target liver cells, a H_2_O_2_‐reactive substrate bis‐(2,4,5‐trichloro‐6‐(pentyloxycarbonyl)phenyl)oxalate (CPPO) for chemiluminescence, and IR775COOH for ONOO^−^ detection (Figure 4c). In the absence of RONS, FRET yields dual emissions at ∼680 and ∼820 nm. The presence of ONOO^−^ abolishes FRET to produce a surge in 680 nm fluorescence (Figure 4d, *top*) and H_2_O_2_ triggers CRET‐driven NIR chemiluminescence (Figure 4d, *bottom*). Since fluorescence and chemiluminescence are optically independent pathways, ONOO^−^ and H_2_O_2_ signals can be read out orthogonally. This capability can help identify specific redox‐driven mechanisms of drug‐ or disease‐induced injury, where pinpointing the responsible oxidant is more informative than measuring overall RONS levels [77]. Under in vivo conditions, CF‐SPN rapidly resolved acetaminophen‐induced oxidative stress, with chemiluminescence rising within minutes (Figure 4e, *left*) followed by an increase in fluorescence (Figure 4e, *right*). SPN‐based nanosensors show a limit of detection of ∼10 and ∼5 nM for ONOO^−^ and H_2_O_2_, respectively, which enables early detection of redox imbalance [78]. Conjugated polymer nanosensors provide a powerful platform for real‐time in vivo imaging of drug‐induced toxicity with dual‐readout capability, high sensitivity to distinct RONS, and prolonged blood circulation.

##### 2.1.2.3 Hydrogen Sulfide (H_2_S)

Hydrogen sulfide (H_2_S) is another key signaling mediator that helps maintain physiological homeostasis [79, 80, 81]. To optically detect H_2_S, Wu et al. synthesized a conjugated electrochromic molecule, dicationic 1,1,4,4‐tetraarylbutadiene (EM 1^2+^), which can be selectively reduced by H_2_S (Figure 5a) [82]. Upon reduction, EM 1^2+^ is converted into a colorless diene, eliminating its broad visible‐NIR absorption. This transformation disrupts intraparticle FRET and restores fluorescence as a turn‐on optical readout for H_2_S. Although this fluorescence‐based strategy enables selective H_2_S detection, its in vivo performance was limited by a relatively low signal‐to‐background ratio (SBR) of ∼3.5, primarily because the continuous excitation produced tissue autofluorescence and photon scattering that elevated the background signal.

**FIGURE 5:**
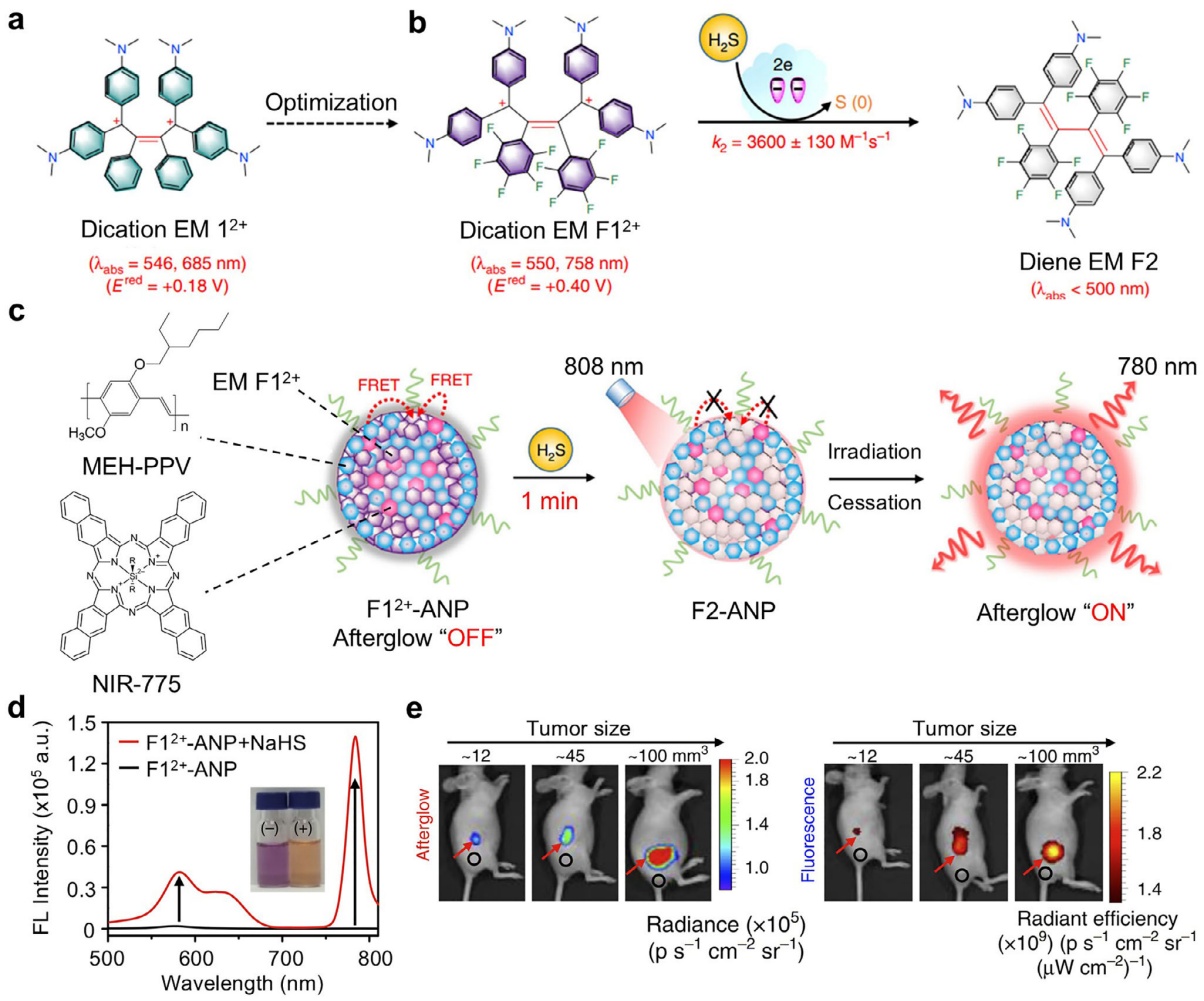
Conjugated polymer nanoparticles for cellular H_2_S detection. (a) Molecular design of EM 1^2+^, (b) EM F1^2+^, and the reduction pathway leading to EM F2. (c) Preparation of the afterglow nanoprobe (ANP), F1^2+^‐ANP, and its H_2_S‐induced luminescence mechanism. The probe encapsulates EM F1^2+^, MEH‐PPV, and NIR775 within PEG to yield the “afterglow‐off” state. Reduction by H_2_S removes FRET‐based quenching and restores fluorescence and afterglow. (d) Fluorescence intensity spectra of F12^+^‐ANP before and after H_2_S activation. Inset: images of the solutions. (e) In vivo afterglow (left) and fluorescence (right) imaging of HepG2 tumors of increasing size 12 h after hepatic‐tumor‐targeting F1^2+^‐ANP injection. Larger tumors exhibit progressively stronger NIR afterglow and fluorescence signals. Panels (a–e) adapted from Ref. [83] under the CC BY 4.0 license. Copyright 2020, The Author(s).

EM 1^2+^ was further optimized by introducing 1,1,4,4‐tetra(pentafluorophenyl)butadiene (EM F1^2+^) (Figure 5b) [83]. With the incorporation of electron‐withdrawing pentafluorophenyl substituents, EM F1^2+^ exhibits red‐shifted absorption in the NIR region with accelerated H_2_S reduction kinetics and enhanced photoluminescence quenching efficiency. Nanoparticles comprised of EM F1^2+^, poly[2‐methoxy‐5‐(2‐ethylhexyloxy)‐1,4‐phenylenevinylene] (MEH‐PPV), and silicon 2,3‐naphthalocyanine bis(trihexylsilyloxide) (NIR775) are non‐fluorescent in their native “OFF” state. Upon H_2_S‐induced reduction, EM F1^2+^ converts to its diene form (EM F2) that eliminates spectral overlap with the emissive components, suppresses FRET, and restores bright fluorescence (Figure 5c). This fluorescence turn‐on is sufficiently large to be observed by the naked eyes (Figure 5d). Moreover, EM F1^2+^ exhibits afterglow emission, in which luminescence persists for tens of minutes after excitation due to the gradual release of stored energy [84]. This enables signal acquisition even after removal of the excitation source. Unlike conventional fluorescence in EM 1^2+^, afterglow imaging decouples excitation and signal acquisition, thereby virtually minimizing excitation‐induced tissue autofluorescence and scattering background. An SBR of ∼72 was achieved for detecting endogenous H_2_S in HepG2 tumor‐bearing mice, which further increased to ∼134 with H_2_S level or the tumor size (Figure 5e). In addition, EM F1^2+^ NPs possess a therapeutic function. Following H_2_S activation, NPs in the excited state generate singlet oxygen within H_2_S‐rich tumor microenvironments that can lead to selective cytotoxicity toward tumor cells. Since the development of such systems, conjugated polymer NPs have emerged as a theranostic platform that combines optical imaging with photodynamic or photothermal therapy. We refer readers to other reviews regarding recent advances in this area [85, 86, 87].

Overall, conjugated polymers provide a versatile platform for optical recording of cellular signals by converting biological activity into measurable colorimetric or spectroscopic changes. Their structural tunability supports diverse optical detection mechanisms. Nevertheless, key challenges including maintaining long‐term stability in biological environments and selectively distinguishing among chemical and electrical signals remain. Advances in molecular and biointerface design of conjugated polymers are expected to further enable real‐time, noninvasive, and high‐throughput optical detection.

### 2.2 Gels and Elastomers

Polymer gels and elastomers are both polymer networks, but they differ in solvent content and elastic response. Gels are solvent‐swollen networks whose mechanics are strongly influenced by solvent‐network interactions, whereas elastomers are solvent‐free and their elasticity arises mainly from chain entropy [43, 44]. Polyacrylamide (PAAm) hydrogels and polydimethylsiloxane (PDMS) elastomers are the two most widely used substrate materials for cell mechanotransduction studies [92, 93]. PAAm hydrogels show nearly linear elastic behavior over a broad kilopascal stiffness range (∼1–240 kPa) [94, 95, 96]. In comparison, PDMS elastomers can reach substantially higher moduli (∼3 kPa to ∼2 MPa) [97, 98] without additional monomer modification. Together, these modulus ranges span the physiologically relevant mechanical regimes of diverse cell types (Figure 6a). This includes brain‐like soft substrates for neurons (∼1 kPa) [99, 100], intermediate stiffness for cardiomyocytes (∼10 kPa) [90, 101], and stiff matrices for bone cells (∼100 kPa to ∼1 MPa) [102, 103]. Beyond tunable stiffness, PAAm and PDMS are biocompatible, optically transparent, and amenable to surface functionalization [91, 104, 105, 106]. These properties enable tunable cell culture substrate mechanics and optical interrogation of cell mechanotransduction under well‐controlled conditions.

**FIGURE 6:**
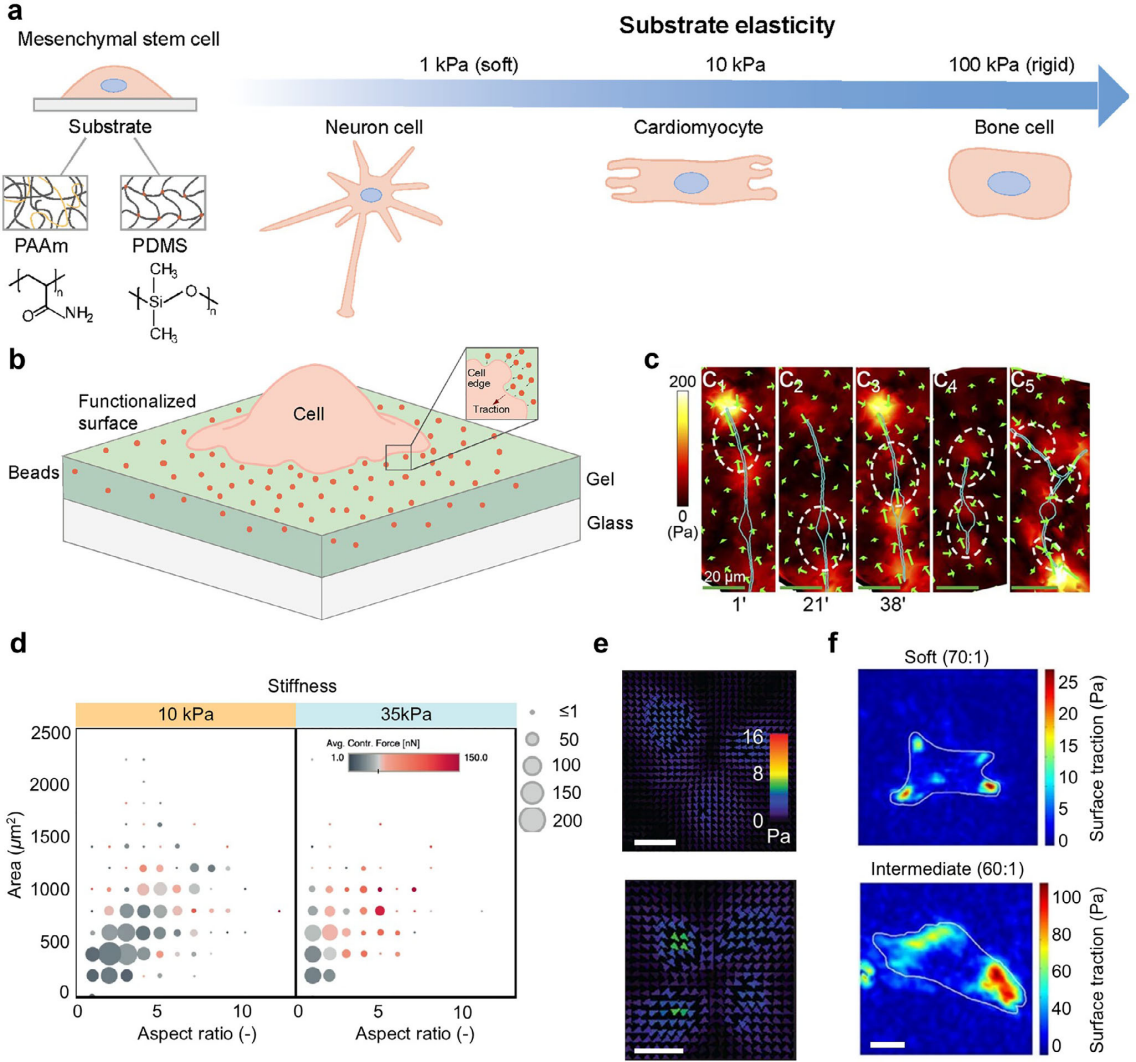
PAAm‐ and PDMS‐based substrates for traction force microscopy. (a) Representative schematic of substrate elasticity‐dependent lineage specification in mesenchymal stem cells, where soft (∼1 kPa), intermediate (∼10 kPa), and stiff (∼100 kPa) matrices promote neuronal‐, myogenic‐, and osteogenic‐like differentiation, respectively. (b) Schematic of TFM setup. (c) Traction stress maps of neurons on PAAm substrates. Time‐resolved maps show the evolution of contraction centers within a single granule cell (C_1_–C_3_) and their coexistence and spatial localization of along neurites (C_4_–C_5_). (d) Population‐level analysis of cardiomyocyte contractility on PAAm substrates with different stiffness shows enhanced force generation on stiffer substrates (35 kPa) and reduced population of highly spread cells. (e) Traction force maps of cardiomyocytes on PAAm substrates show localized and directionally organized contractile forces. (f) Traction stress maps of human bone marrow‐derived mesenchymal stem cells on PDMS substrates with varying stiffness show higher and more localized stresses on stiffer substrates. Scale bar = 25 µm. Schematics in panels (a and b) created using Adobe Illustrator. Panel c reproduced with permission from Ref. [88] under the CC BY‐NC‐SA 3.0 license. Copyright 2015, Jiang et al. Panel (d) adapted from Ref. [89] under the CC BY 4.0 license. Copyright 2024, The Author(s). Panel (e) adapted from Ref. [90]. Panel (f) reproduced from Ref. [91].

#### 2.2.1 Biomechanical Signal Detection

The mechanical properties (i.e., stiffness and viscoelasticity) of ECM critically influence cellular behavior [107, 108, 109]. Understanding force‐based communications between cells and the extracellular matrix (ECM) is crucial for elucidating how mechanical signals regulate cell fate [110, 111, 112]. Polymer gels and elastomers can serve as engineered substrates with tunable ECM‐like mechanics to enable quantitative measurements of cell responses to different extracellular environments.

##### 2.2.1.1 Traction Force Microscopy (TFM)

Traction force microscopy (TFM) commonly uses polymer substrates, such as PAAm or PDMS, embedded with fluorescent beads as markers to map cell‐generated traction forces (Figure 6b) [113]. As cells adhere to the substrate, they displace the embedded beads, and these bead displacements can be optically recorded and computationally reconstructed into traction stress maps based on the substrate modulus and established elastic models. The conceptual basis of TFM dates back to the 1980 study by Harris et al. where cells cultured on thin silicone rubber substrates generated visible wrinkles in response to contractile forces [114]. Since then, TFM has evolved into a quantitative approach for mapping the spatial distribution of cellular stresses transmitted to soft substrates [97].

###### 2.2.1.1.1 PAAms in TFM

PAAms have been widely used in TFM because of their linear elastic behavior [115]. Their optical transparency allows integration with fluorescence imaging to simultaneously visualize focal adhesion [116] and cytoskeleton stress fibers [117]. In addition, their stiffness can be precisely tuned to mimic diverse biological tissues to control cell mechanotransduction in parallel with TFM measurements [95, 118]. Beyond their mechanical and optical advantages, PAAm hydrogels can be functionalized with ECM proteins or adhesive ligands, making them well‐suited for TFM studies of adherent cells [119, 120].

Using PAAm substrates, Jiang et al. mapped traction stress fields in migrating neurons [88]. Time‐resolved traction maps revealed multiple contraction centers in the neuronal leading and trailing processes, whose dynamics correlated with soma translocation (Figure 6c). These centers emerged, disappeared, and shifted over time, indicating dynamic reorganization of traction forces during neuronal migration. Extending PAAm‐based TFM to contractile cells, the Pruitt group employed PAAm substrates to investigate how substrate rigidity and cell geometry regulate the contractile behavior of hiPSC‐derived cardiomyocytes [89, 90, 121]. By fabricating gels that emulate healthy (∼10 kPa) and fibrotic myocardium (∼35 kPa), they demonstrated that increased substrate stiffness enhanced cardiomyocyte contractile strength (Figure 6d), which establishes a clear link between the mechanical microenvironment and cardiac force generation. They also used TFM maps to illustrate the spatial distribution of cardiomyocyte‐generated forces on compliant PAAm substrates (Figure 6e) [90].

###### 2.2.1.1.2 PDMS in TFM

Complementing PAAm hydrogels, PDMS elastomers provide mechanically stable and durable substrates for TFM, particularly in experiments that require long‐term culture, repeated measurements, or device integration [105]. Unlike hydrogel‐based substrates, PDMS forms nonporous and relatively impermeable elastomeric surfaces that limit biochemical diffusion into the substrate and help maintain stable boundary conditions during TFM measurements [105]. These properties allow PDMS to be used for fluorescence‐based force measurements in controlled, device‐compatible cell culture platforms.

Building on these material advantages, PDMS‐based TFM has been used to examine stiffness‐dependent force generation in mechanosensitive cells. Using PDMS substrates with controlled stiffness, Razafiarison et al. quantified traction stresses generated by human bone marrow‐derived mesenchymal stem cells (Figure 6f) [91]. Cells generated higher and more localized traction stresses on stiffer PDMS substrates, consistent with stiffness‐dependent trends reported for PAAm‐based systems. PDMS‐based TFM has also been used to examine how biochemical cues regulate cellular force generation. Yoshie et al. adapted PDMS‐based TFM platform to a parallelized 96‐well plate format to investigate cell contractility changes associated with epithelial‐to‐mesenchymal transition (EMT) [122]. They quantified epithelial cell traction forces under varying concentrations of the soluble factor TGF‐β with high throughput. Naha and Driscoll further employed soft PDMS substrates (∼10 kPa) coated with distinct ECM proteins to study how traction forces in nucleus pulposus cells during disc degeneration evolves [123]. Their traction maps showed striking ECM‐specific variations, with fibronectin coatings inducing highly localized and elevated stress patterns.

The durability and processability of PDMS further facilitate the integration of TFM with dynamic and device‐based culture environments. Das et al. integrated PDMS‐based TFM platform with microfluidic flow channels to assess stress distributions in adherent mouse fibroblast L929 cells under static and flow conditions [124]. This design allowed fluid shear and incubation time to be examined as dynamic regulators of cellular traction forces. Device‐integrated PDMS platforms also expand TFM beyond static substrate‐based measurements toward force mapping under more complex mechanical environments.

PAAm and PDMS substrates have established TFM as a quantitative optical approach for mapping cellular forces under controlled extracellular conditions. Nevertheless, both systems are simplified ECM models that capture cell‐generated forces in the 2D space. This limitation should be considered when interpreting traction maps, particularly when translating results from in vitro force measurements to in vivo conditions. From a materials perspective, TFM will benefit from substrates that better mimic tissue viscoelasticity and ligand presentation, together with models that enable more physiologically relevant analysis and reconstruction of cell‐generated forces.

###### 2.2.1.1.3 Recent Advances in TFM

Recent methodological advances have addressed key limitations of conventional TFM by extending force measurements from two to three dimensions. To better capture the three‐dimensional (3D) nature of traction forces, Legant et al. developed multidimensional TFM that resolves both in‐plane (shear) and out‐of‐plane (normal) cell traction stresses (Figure 7a) [125]. Both shear and normal tractions are found to be localized at the cell periphery with maximum shear stress (∼550 Pa) occurring near the termini of actin stress fibers. Negligible stresses were found in perinuclear and nuclear regions.

**FIGURE 7:**
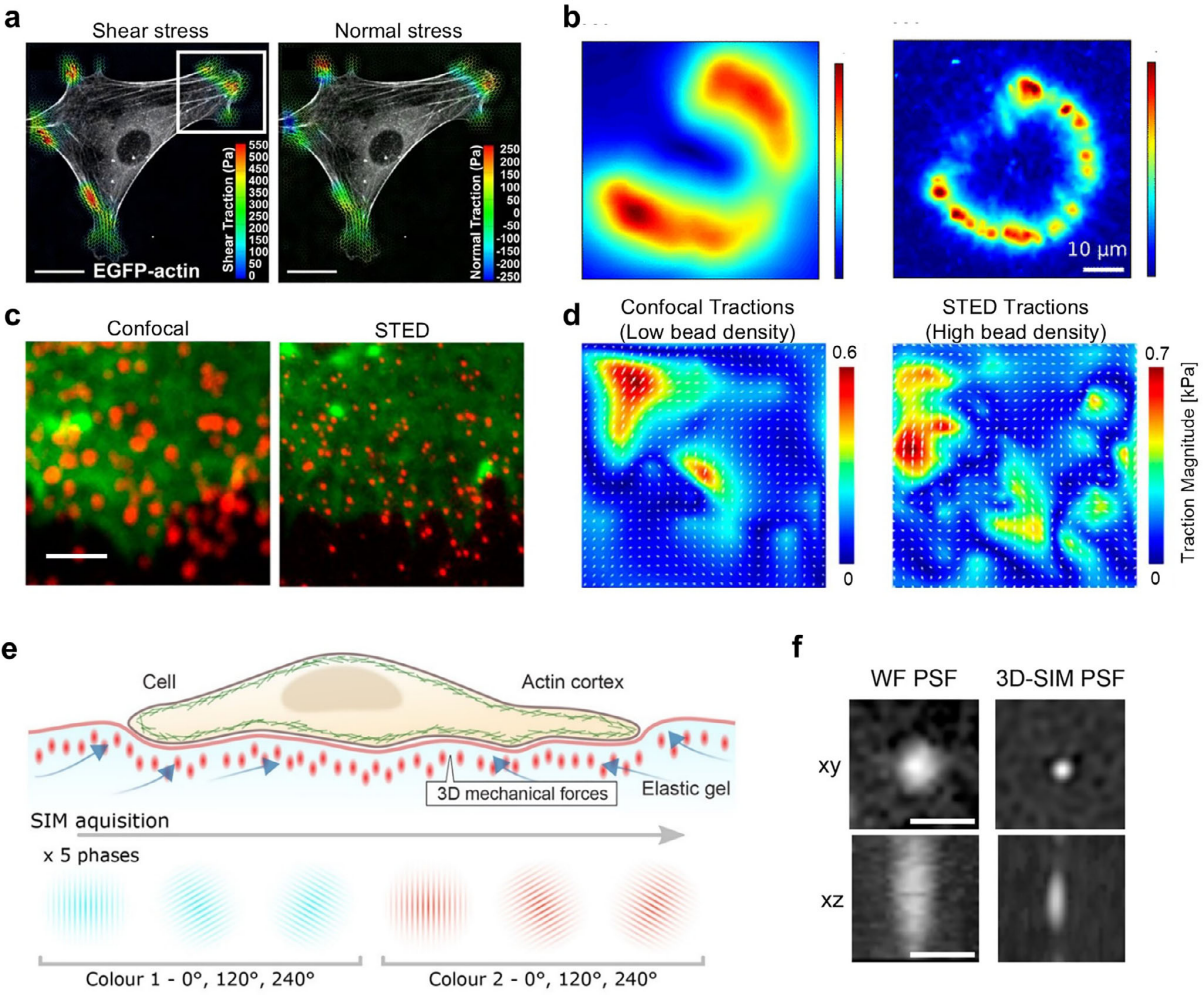
Recent advancements in TFM. (a) Shear (left) and normal (right) traction stresses in mouse embryo fibroblasts (MEFs) expressing EGFP‐actin. The two components reveal distinct spatial patterns of in‐plane and out‐of‐plane forces. Scale bars = 10 µm. (b) Comparison of traction force maps obtained by confocal TFM (left) and FBSR‐TFM (right). FBSR‐TFM improves bead‐tracking precision and spatial resolution. Scale bar = 10 µm. (c) Confocal and STED images of spreading cells on PAAm substrates (F‐actin, green; embedded fluorescent beads, red). STED imaging resolves the beads more clearly. Scale bar = 2 µm. (d) Reconstructed traction fields from confocal low‐density bead tracking and STED high‐density bead tracking. STED‐based reconstruction provides higher‐resolution traction maps. (e) Schematic of 3D structured illumination microscopy (3D‐SIM) combined with 3D‐TFM for mapping cell‐generated forces within elastic gels. (f) Fluorescent bead images acquired by wide‐field microscopy and 3D‐SIM with improved resolution in both lateral (*xy*) and axial (*xz*) directions. Scale bars = 0.5 µm. Panel (a) adapted from Ref. [125]. Panel (b) adapted from Ref. [126] under the CC BY 4.0 license. Copyright 2020, American Chemical Society. Panels (c and d) adapted with permission from Ref. [127] under the CC BY 4.0 license. Copyright 2016, American Chemical Society. Panels (e and f) adapted from Ref. [128] under the CC BY 4.0 license. Copyright 2016, American Chemical Society.

Another major direction is to improve the spatial resolution and reconstruction accuracy of traction forces. Stubb et al. developed a fluctuation‐based super‐resolution TFM (FBSR‐TFM), which applies fluorescence fluctuation analysis to achieve sub‐diffraction traction mapping with improved signal‐to‐noise ratio (Figure 7b) [126]. This approach enabled high‐resolution quantification of local traction force heterogeneity within adhesion complexes. Colin‐York et al. further introduced stimulated emission depletion‐based super resolved TFM (STED‐STFM) [127, 129]. This method achieved approximately five‐fold higher spatial resolution than conventional TFM with improved traction retrieval accuracy (Figure 7c,d). The same group later integrated 3D structured illumination microscopy with TFM (3D‐SIM‐TFM) to enable volumetric force measurements in living cells with both improved lateral (*xy*) and axial (*xz*) resolution (Figure 7e,f) [128].

##### 2.2.1.2 Other Force Microscopy Methods

TFM has been a benchmark technique for quantifying cell‐substrate mechanical interactions, but its quantitative precision depends on inverse elasticity modeling and the computational traction reconstruction [132]. To complement these limitations, other materials‐based optical force assays have been developed to convert cell‐generated forces into more direct, localized, or scalable readouts.

Gibbons et al. reported a platinum‐coated PDMS diffraction grating that converts cardiomyocyte contraction into real‐time changes in diffraction intensity (Figure 8a) [130]. Neonatal rat cardiomyocytes were cultured on fibronectin‐functionalized gratings, and epinephrine stimulation increased their beating frequency from 0.6 bps to 1.1 bps (Figure 8b). This grating‐based platform provides a scalable optical readout of contractile activity, making it useful for screening dynamic cardiomyocyte responses. But it primarily reports collective contractility rather than spatially resolved traction maps as in TFM. Tan et al. introduced arrays of elastomeric silicone (PDMS) microposts that function as independent microscale cantilevers (Figure 8c) [131]. Cell traction forces bend individual posts, and the local force on each post can be calculated from its lateral deflection. By assembling post‐wise forces into a vector map, this platform directly visualizes subcellular traction patterns (Figure 8d). A positive correlation between focal adhesion size and local force magnitude was also observed (Figure 8e). Compared with conventional 2D TFM, micropost arrays simplify force reconstruction because each post acts as an individually calibrated force sensor, although their discrete surface topography may influence cell adhesion and force organization. To this end, non‐substrate‐based optical techniques, such as optical tweezers and brillouin microscopy, also offer powerful means to measure cell forces and are reviewed elsewhere [133, 134, 135, 136, 137].

**FIGURE 8:**
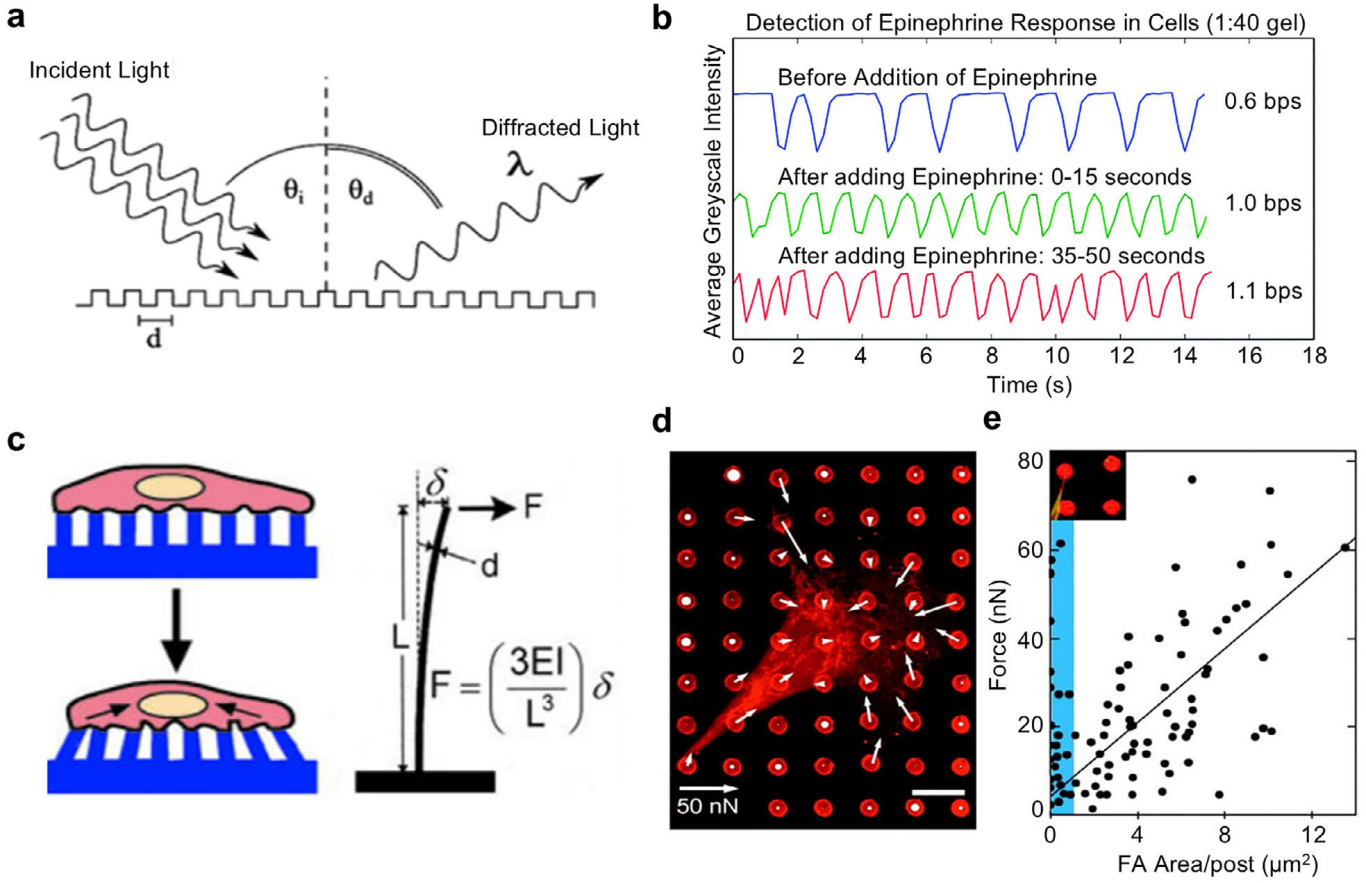
Other materials‐based force microscopy methods. (a) PDMS diffraction grating for optical detection of cellular contractility. (b) Optical readouts of cardiomyocyte beating, showing increased frequency (from 0.6 bps to 1.1 bps) after epinephrine stimulation. (c) Micropost array traction assay. Schematic of elastomeric post deflection under cell‐generated force. (d) Fluorescent image of post‐tip displacements (white arrows). Scale bar = 10 µm. (e) Cell force generated on each post as a function of total focal adhesion area per post. The shaded region (blue) indicates the adhesions smaller than 1 µm2. Panels (a and b) adapted with permission from Ref. [130]. Copyright 2017, Royal Society of Chemistry. Panels (c–e) adapted from Ref. [131]. Copyright 2003, National Academy of Sciences.

## 3 Liquid Crystals

Liquid crystals (LCs) are unique materials that exist in a state between solid and liquid [138]. In the nematic phase, LC molecules lack long‐range positional order but remain roughly aligned along their long axes. Smectic phases often form at lower temperatures with molecules organized into distinct layers that can slide past one another. Smectic phases include smectic A (SmA) and smectic C (SmC). In SmA, molecules align on average with the layer normal, whereas in SmC, they are tilted relative to it. A particularly important variant is the chiral smectic C phase (SmC*, the asterisk denotes chirality). In SmC*, chirality causes the molecular tilt direction to rotate progressively between layers, forming a helical superstructure along the layer normal. Each LC phase exhibits distinct optical and electrical anisotropy, including characteristic birefringence arising from its molecular organization.

### 3.1 Deformed‐Helix Ferroelectric Liquid Crystals

In the SmC* phase, molecular tilt and chirality break inversion and mirror symmetry. This allows spontaneous polarization and ferroelectric switching that is absent in nonchiral SmA and SmC phases. Most ferroelectric liquid crystals (FLCs) are in the SmC* phase. Conventional FLC devices typically use an unwound helix and switch between two stable states under an electric field, whereas deformed‐helix ferroelectric liquid crystals (DHF‐LCs) retain a short‐pitch helix that deforms continuously [139]. With a subwavelength helix pitch, DHF‐LCs offer smooth optical modulation at low voltages and sub‐millisecond electro‐optical responses [140, 141]. DHF‐LCs are usually multicomponent mixtures comprising molecules with different structures (Figure 9a) [141, 142].

**FIGURE 9:**
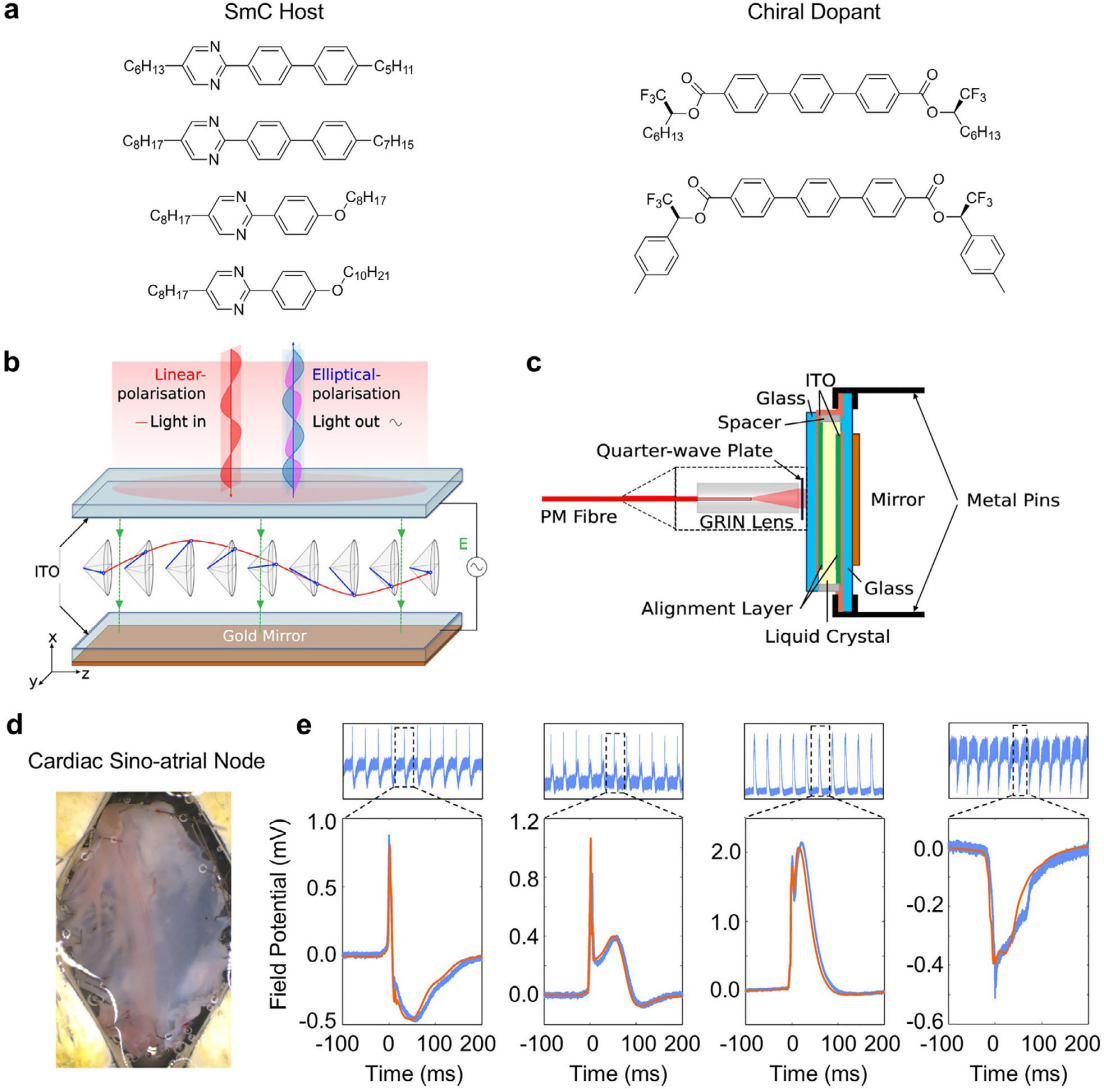
Recording bioelectric signals using liquid crystal optrode. (a) Representative molecular components of DHF‐LC formulations. The mixture typically combines rod‐like SmC host mesogens with chiral dopants. (b) Operation principle of electro‐optical transducers that use DHF‐LCs. (c) Cross‐section of a typical liquid‐crystal‐based optrode for electrophysiological studies. Polarization maintaining (PM) fiber transmits polarized incoherent white light through a gradient refractive index (GRIN) lens, which expands the beam into the transducer. The light passes through the liquid crystal layer, reflects off the back mirror, and is recaptured by the PM fiber. (d) Image of cardiac sino‐atrial node tissue. (e) Spontaneous field potentials from rabbit sinoatrial nodes and adjacent right atrial pectinate muscle recorded using optrode (blue) and electrode (orange). The zoomed‐in panel shows an average of 50 waveforms. Panels (b–e) adapted with permission from Ref. [143]. Copyright 2022, IOP Publishing Ltd.

#### 3.1.1 Bioelectric Signal Detection

The Lovell group used DHF‐LCs to build optoelectronic transducers, called “optrodes,” for bioelectric signal recording [143, 144, 145]. Before application, LCs typically require alignment including photoalignment with light‐sensitive molecules (e.g., azobenzene sulfuric dye), polymer rubbing with a polyimide alignment layer, and oblique evaporation with a thin silicon dioxide film [146]. In this work, DHF‐LCs were aligned with a rubbed planar‐aligning polyimide layer (60 nm) on the inner ITO surfaces. A gold mirror was attached to the outer face of one ITO‐glass substrate. Polarized 1550 nm light delivered using a polarization‐maintaining fiber was reflected back through the DHF‐LC layer by the mirror and coupled into the same fiber, which acted as both polarizer and analyzer (Figure 9b,c).

In the absence of an electric field, DHF‐LCs orient in a helical structure with the axis parallel to the substrate plane. When an electric field perpendicular to the helical axis is applied, the LC dipole moments align with the field and deform the helix. This deformation rotates the optical axes by an angle proportional to the applied field strength (e.g., <0.1 V µm^−1^). With a linear polarizer on the front substrate and a mirror on the back, incident light passes through the LC and polarizer twice, such that its intensity can be modulated by the applied electrical field. Using this design, the authors demonstrated optrode recordings of field potentials from rabbit sinoatrial node tissue (Figure 9d). The recorded signals showed good agreement with conventional electrode recording, achieving a signal‐to‐noise ratio of 6.6 dB at 11.1 kHz recording bandwidth (Figure 9e) [143].

### 3.2 Nematic Liquid Crystals

#### 3.2.1 Biochemical Signal Detection

Nematic liquid crystals (nematic LCs) can be used in biochemical detection and imaging. Aligned LCs are typically doped with recognition molecules (e.g., lipids, antibodies, nanoparticles). When recognition molecules interact with target analytes, LC alignment is disrupted and produces detectable optical signals that can even be used for chemical mapping [147]. As the current review focuses on detecting biological signals with live cells or tissues, we refer the readers to other comprehensive reviews for small molecule chemical sensing using LCs [148, 149].

Liquid crystals possess several advantages that make them attractive materials for optically detecting cellular signals. The manufacturing technologies developed for the display industry allows low‐cost device fabrication. The long‐range orientational order of LCs allows local interfacial perturbations to trigger collective molecular reorientation. This provides intrinsic optical signal amplification and rapid signal transduction in a label‐free manner [150], which are beneficial for detecting fast cellular signals. However, practical limitations remain for LCs. Because they are fluid‐like, they generally require enclosed cell architectures. This complicates integration into wearable or implantable devices. Their high sensitivity also makes them vulnerable to perturbations such as temperature fluctuations or nonspecific interfacial interactions, which can reduce measurement reproducibility [150]. Improving detection stability and specificity without sacrificing sensitivity are key directions for future research [151].

## 4 Carbon‐Based Materials

Carbon‐based materials have emerged as a versatile platform for optical biosensing because their extended sp^2^‐hybridized electronic structures can convert local electrical or chemical perturbations into measurable optical readouts [152, 153]. This is advantageous for detecting weak, spatially heterogeneous cellular signals.

### 4.1 Graphene and Derivatives

Graphene is a single layer of carbon atoms arranged in a 2D honeycomb lattice, with high carrier mobility, atomic‐scale thickness, and a gate‐tunable electronic structures [154]. Changes in its Fermi level, interfacial charge density, and local dielectric environment can modulate its optical absorption and reflectance [155], making graphene attractive for optical detection of bioelectric activities. Graphene derivatives, particularly graphene oxide, contain oxygen‐bearing groups that improve aqueous dispersibility, allow biofunctionalization, and enable fluorescence‐based detection of biochemical signals through efficient quenching [156].

#### 4.1.1 Bioelectric Signal Detection

Graphene shows field‐sensitive optical transitions such that an applied electrical potential can modulate its optical absorption via Pauli blocking of interband transitions (Figure 10a) [155]. Leveraging this unique property, Horng et al. demonstrated the critically coupled waveguide‐amplified graphene electric field (CAGE) platform [158]. This platform critically couples light into the graphene layer using a prism optical waveguide (Figure 10b,c). Under such conditions, incident light is almost entirely absorbed by the graphene layer to maximize its interaction with graphene. Therefore, small changes in graphene absorption induced by electric fields can produce large changes in reflectivity. To assess the performance of the CAGE sensor, the authors characterized the voltage detection sensitivity as a function of the applied gate voltage to graphene and compared experimental results with theoretical simulations. The experiments matched well with the modeling prediction. The GAGE sensor reaches a maximum voltage sensitivity of 1.2% optical signal change (Δ*R*/*R*) per millivolt at a graphene gate voltage of +0.53 V (Figure 10d). This corresponds to an experimental voltage detection limit of ∼15 µV, which represents an over 200‐fold improvement in sensitivity relative to conventional graphene optical sensors [158]. Moreover, the CAGE sensor maintained such sensitivity for up to 10 kHz signals with a detection spatial resolution on the order of tens of micrometers. These capabilities allowed capturing rapid and spatially heterogeneous electric fields.

**FIGURE 10:**
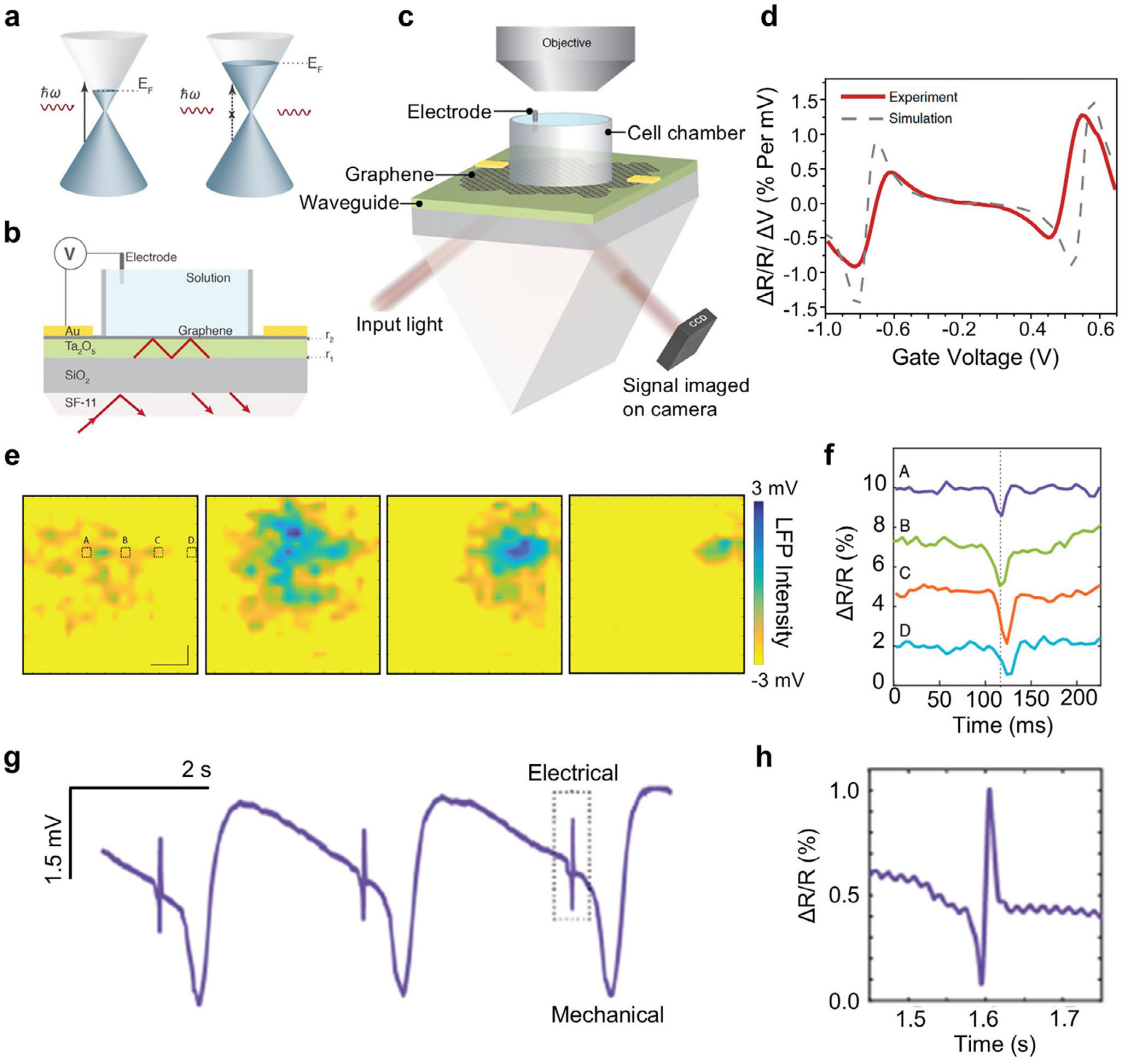
Recording bioelectric signals using graphene optoelectronic devices. (a) Schematic of interband transitions in graphene showing a gate‐induced shift in the Fermi level. Optical modulation is strongest near the Fermi surface, where an external field shifts the Fermi energy and suppresses interband optical absorption through Pauli blocking. (b) Cross‐sectional schematic of the CAGE sensor. The two interfaces, characterized by reflection coefficients *r*_1_ and *r*_2​_, form an optical cavity. (c) Schematic of the CAGE system. (d) Experimental (solid red line) and simulated (dashed gray line) voltage sensitivity of the CAGE sensor, defined as (Δ*R*/*R*)/Δ*V*, as a function of gate voltage. (e) CAGE images capture the propagation of extracellular field potentials across spontaneously beating heart tissue within an approximately 2 mm^2^ field‐of‐view. Time lapse between frames = 5 ms. Scale bar = 400 µm. (f) Signals at locations A–D in (e) are plotted as time series to show delayed electrical responses as they traverse the tissue. (g) CAGE recording traces of spontaneous field potentials and mechanical contractions in heart tissue. (h) Zoomed‐in view of the extracellular field potential in the dashed box in (g). Panels (a–c) and (e–h) adapted with permission from Ref. [157]. Copyright 2021, American Chemical Society. Panel (d) adapted from Ref. [158] under the CC BY 4.0 license. Copyright 2016, The Author(s).

Balch and colleagues employed the CAGE sensor to achieve label‐free optical recording of bioelectric signals from chicken heart tissue [157]. Under the imaging mode (using camera), the CAGE sensor mapped local field potentials from spontaneously beating chicken heart tissue with millivolt sensitivity over a ∼2 mm^2^ field of view and a temporal resolution of ∼5 ms (Figure 10e). The sequential propagation of extracellular field potentials across the tissue was also imaged. This allowed the conduction velocity of bioelectric signals to be determined (14 cm/s) without signal averaging (Figure 10f). Under the recording mode (using photodetector), the time‐resolved recording traces further distinguished rapid electrical spikes (∼1.36 mV) from slower mechanical contractions (Figure 10g,h). The CAGE sensor provides a stable optical readout of bioelectric activities and direct visualization of the propagating electric field using graphene as an optical probe. Nevertheless, achieving optimal voltage sensitivity with the CAGE sensor requires gate biases of several hundred millivolts. For single‐cell recordings, the effects of such biasing on cell compatibility require further evaluation.

#### 4.1.2 Biochemical Signal Detection

Graphene derivatives, such as graphene oxide (GO), can act as photoluminescence emitters and be used as efficient quenchers for FRET‐based biochemical signal detection [159, 160]. GO‐based aptamer platforms have been applied to sense and monitor intracellular adenosine triphosphate (ATP) levels [161, 162, 163, 164]. These sensing platforms generally rely on a fluorescence “OFF/ON” transduction mechanism such that GO nano sheets serve as both an efficient fluorescence quencher and a carrier for intracellular delivery of aptamer probes (Figure 11a). In the absence of ATP, fluorophore‐labeled aptamers adsorb onto the GO surface through π–π stacking interactions, bringing the fluorophores close to GO and quenching their fluorescence. When intracellular ATP binds to the aptamers, the resulting conformational change weakens their interaction with GO and restores the quenched fluorescence. This mechanism can further be extended to multiplexed detection of ATP and other nucleoside triphosphates such as guanosine triphosphate (GTP) using target‐specific aptamers labeled with spectrally distinct fluorophores (Figure 11b) [162]. As shown in Figure 11c, confocal fluorescence images demonstrate simultaneous, target‐specific detection of intracellular ATP and GTP using the GO‐aptamer platform [162].

**FIGURE 11:**
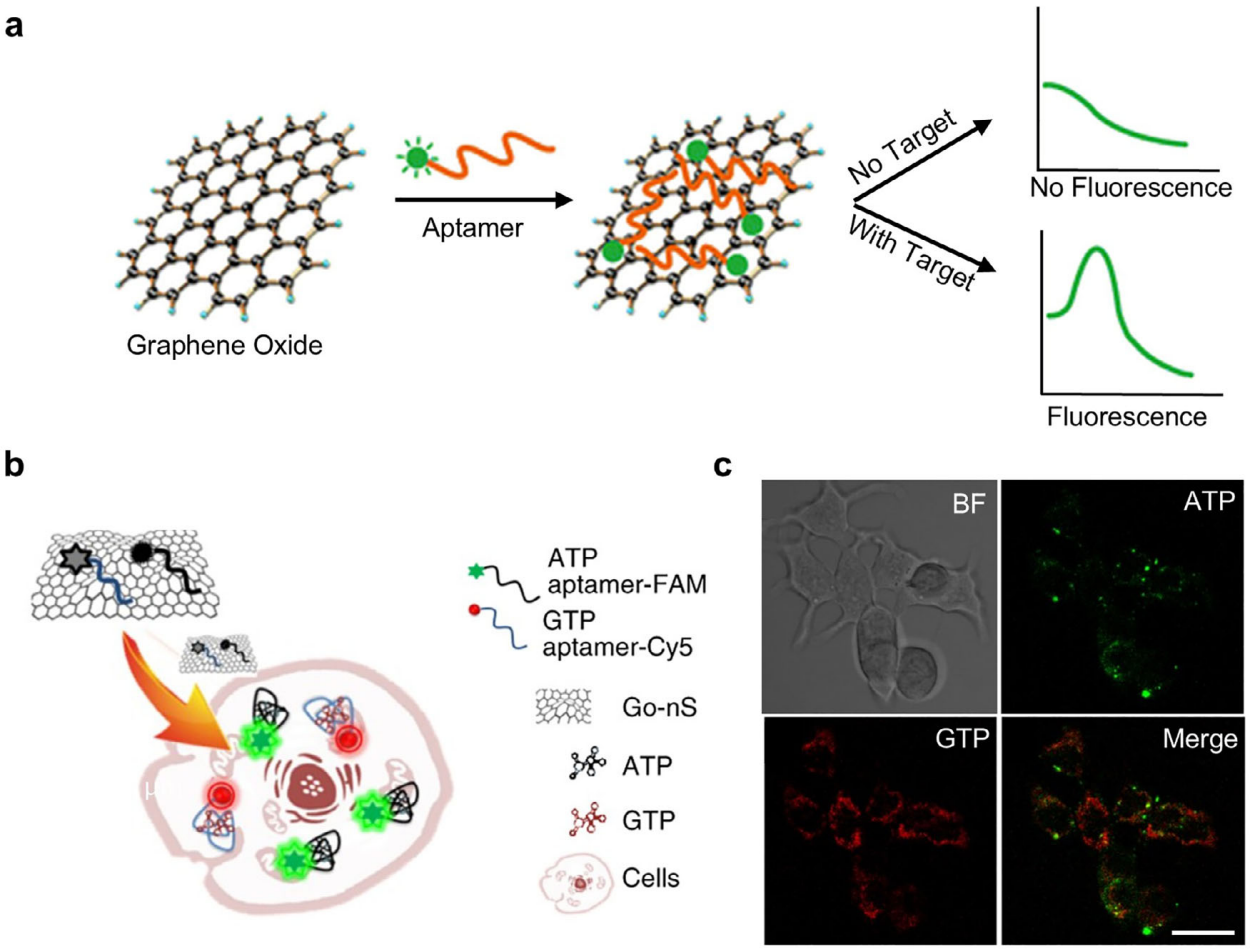
Live‐cell chemical sensing using aptamer‐functionalized graphene oxide. (a) Schematic of the fluorescence “OFF/ON” mechanism of the aptamer/GO nanocomplex. Fluorophore‐labeled aptamers are quenched on GO and released upon target binding that results in fluorescence recovery. (b) In situ intracellular probing of ATP and GTP using GO nanosheets carrying fluorophore‐labeled ATP and GTP aptamers. (c) Bright field and confocal fluorescence images demonstrating simultaneous visualization of ATP and GTP. Scale bar = 10 µm. Panel a adapted from Ref. [173] under the CC BY 4.0 license. Copyright 2018, The Author(s). Panels (b and c) adapted with permission from Ref. [162]. Copyright 2014, Springer Nature.

Graphene and GOs can further be functionalized to optically detect ions, proteins, and nucleic acids, through specific recognition chemistries and surface interactions in cell‐free systems [165, 166, 167]. Graphene quantum dots (GQDs) have also been developed as fluorescent probes for bioimaging due to their tunable fluorescent emission, high photostability, and low cytotoxicity. We refer the readers to reviews elsewhere for these discussions [168, 169, 170, 171, 172].

However, several challenges remain for optical detection of cellular signals using graphene and its derivatives. Graphene's intrinsic optical modulation is modest and often requires photonic enhancement or external biasing. This increases device complexity and complicates biological integration. Optical detection signals from graphene oxide are typically indirect and depend strongly on surface functionalization. Future research should focus on enhancing intrinsic optical detection sensitivity and developing stable, selective surface chemistries for different analytes.

### 4.2 Single‐Walled Carbon Nanotubes

Single‐walled carbon nanotubes (SWCNTs) are “rolled‐up graphene,” where carbon atoms are arranged in a hexagonal monolayer to form cylindrical structures [174, 175]. A central principle for their use in chemical sensing is Corona Phase Molecular Recognition (CoPhMoRe) [176], where polymers adsorbed onto the SWCNT surface can adopt constrained conformations to create a binding pocket for specific analytes (Figure 12a). Upon analyte binding, the SWCNT–polymer interface is perturbed and the excitonic states of the carbon nanotube are modified. This results in measurable photoluminescence intensity changes in the NIR region of the SWCNTs.

**FIGURE 12:**
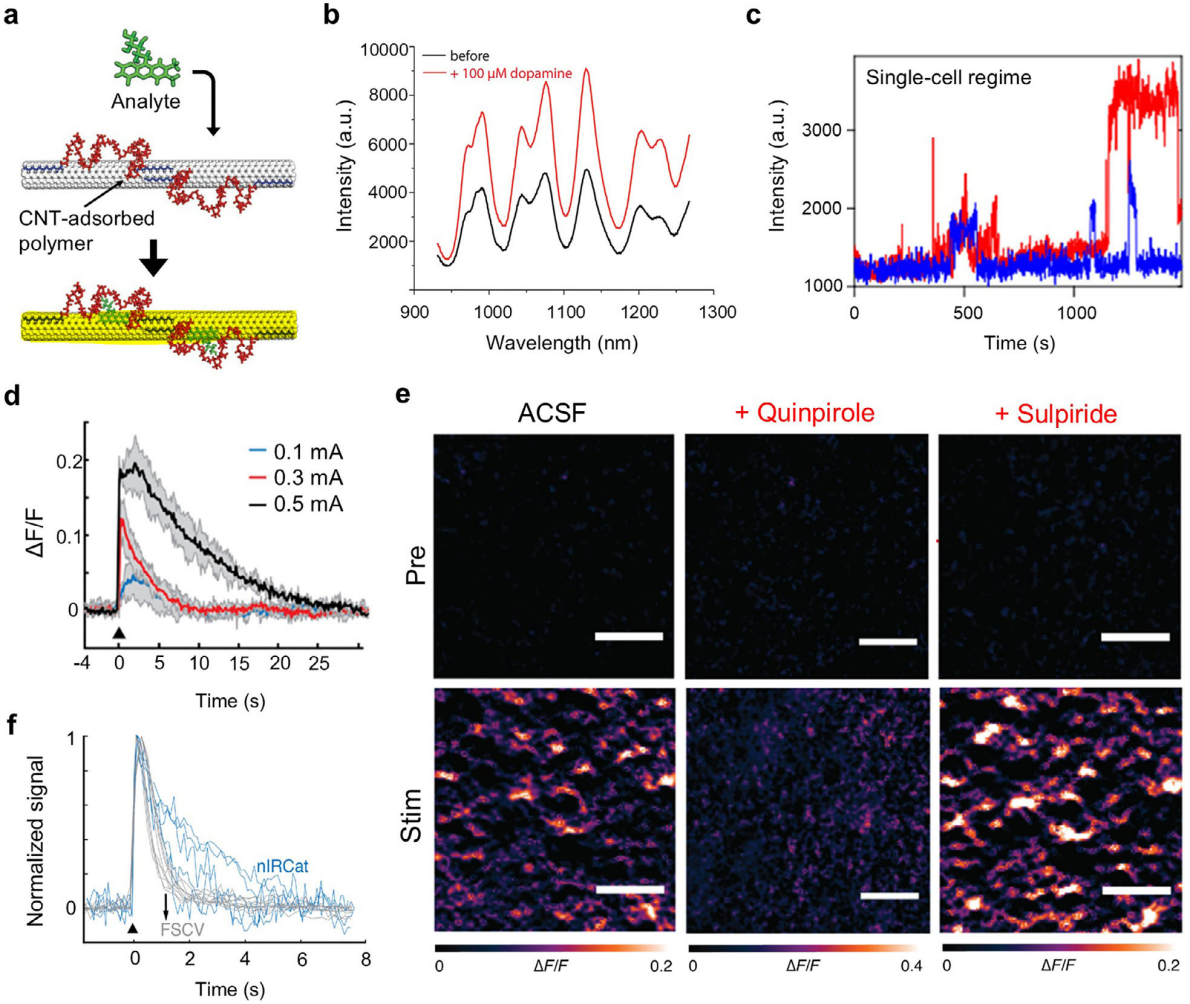
Biochemical signal detection through Corona Phase Molecular Recognition (CoPhMoRe) of SWCNTs. (a) Schematic of CoPhMoRe. The adsorbed polymer forms a recognition site for the target analyte. Analyte binding leads to a change in fluorescence intensity of SWCNT. (b) Dopamine dependence of the NIR fluorescence spectrum of (GT)_15_‐SWCNT in phosphate‐buffered saline (PBS). (c) Time traces of RAP1 protein secretion of agarose‐gel‐immobilized *E. coli* imaged using individual SWCNT nanosensors. (d) Fluorescence intensity change of nIRCats upon single‐pulse electrical stimulation of mouse striatal slices. (e) Fluorescence images of mouse striatal slices with nIRCats before (Pre) and during electrical stimulation (Stim) under three conditions: artificial cerebrospinal fluid (ACSF) as control (left), 1 µM quinpirole (center), and 1 µM sulpiride (right). Color scale represents fractional fluorescence change (Δ*F*/*F)*. Scale bars = 10 µm. (f) Dopamine signal measured using FSCV (gray) and nIRCat (blue). FSCV reports ensemble‐averaged signals, whereas nIRCat reveals heterogeneities in these signals. Panel (a) adapted with permission from Ref. [176]. Copyright 2013, Springer Nature. Panel (b) reproduced with permission from Ref. [177]. Copyright 2014, American Chemical Society. Panel (c) reproduced with permission from Ref. [178]. Copyright 2017, Springer Nature. Panels (d–f) adapted from Ref. [179] under the CC BY‐NC 4.0 license. Copyright 2019, The American Association for the Advancement of Science.

#### 4.2.1 Biochemical Signal Detection

Kruss et al. employed this concept for neurotransmitter detection by using nucleic acid corona [177]. The authors showed that, depending on the chirality of the SWCNT and the sequence of single‐stranded DNA/RNA wrapping around it, the electronic environment of the nanotube surface can be tuned. As a result, polymer‐wrapped SWCNTs exhibit increased NIR fluorescence upon surface binding of catecholamines such as dopamine. In particular, for (GT)_15_ nucleic acid wrapped SWCNT conjugates, the addition of 100 µM dopamine showed a near one‐fold increase in fluorescence (Figure 12b). This optical response corresponds to a dissociation rate constant in the sub‐mM range and a dopamine detection sensitivity of ∼10 nM. When immobilized, single carbon nanotubes displayed up to five‐fold intensity changes, with fluorescence fully reversible upon analyte removal. Landry et al. combined an aptamer‐anchor DNA sequence adsorbed to the SWCNT surface to achieve selectivity for specific proteins [178]. Such design enabled label‐free, single‐molecule level detection of proteins such as RAP1 and HIV integrase. When deployed in microfluidic chambers, arrays of immobilized sensors can detect protein secretion from cell lysates and simultaneously track the secretion dynamics of single *E. coli*, human embryonic kidney 293 (HEK 293) cells and *Pichia pastoris* cells. As shown in Figure 12c, RAP1 protein secretion from agarose gel‐immobilized *E. coli* bacterium can be monitored in real time through stepwise increase in SWCNT fluorescence intensity. These studies demonstrate the advantage of SWCNT nanosensors in achieving ultralow detection limits while maintaining molecular specificity in solution.

Beyene et al. further developed a NIR catecholamine nanosensors (nIRCats) by functionalizing SWCNTs with short (GT)_6_ oligonucleotides [179]. These nanosensors produce up to 24‐fold fractional fluorescence changes (Δ*F*/*F*) in response to dopamine and span a dopamine concentration dynamic range of 10 nM–100 µM. NIRCats also exhibited high selectivity for dopamine and norepinephrine, which overcomes the poor chemical sensitivity of conventional electrochemical probes [180]. Under different stimulation currents, nIRCats optically report electrically evoked dopamine release, as shown by fluorescence transients (Figure 12d). When applied to mouse striatal slices, fluorescence hotspots of ∼2 µm in size are recognized as dopaminergic terminals (Figure 12e, *left*). NIRCats also report dopamine fluorescent signal changes under physiological modulation: the activation of presynaptic D_2_ autoreceptors with quinpirole attenuated the fluorescent transient (Figure 12e, *center*), whereas blockade with sulpiride enhanced it (Figure 12e, *right*). Compared to electrode‐based dopamine detection methods such as fast‐scan cyclic voltammetry (FSCV) which measure ensemble‐averaged signals [181], optical detection using nIRCats capture both the onset and peak release of dopamine with sub‐second temporal resolution. It is also capable of revealing spatial heterogeneity in dopamine clearance across different locations in the tissue (Figure 12f).

Building upon their previous work, Beyene and colleagues further coupled NIR with visible light fluorescence imaging to reveal the relationship between dopamine efflux and the distribution of release‐related proteins of dopaminergic neurons cultured on 2D nIRCat sensor films [182]. This platform achieved synaptic‐resolution imaging to link individual dopamine release hotspots to specific axonal boutons and dendrites. Although nIRCats currently represent one of the most advanced synthetic optical platforms for catecholamine sensing, the recorded kinetics are influenced by analyte diffusion, reversible sensor binding, and dopamine reuptake, which can limit the temporal resolution of detecting rapid phasic bursts during tonic activity. Moreover, efforts toward quantitative measurements of local neurotransmitter concentrations will allow fluorescence signals to more faithfully reflect synaptic release dynamics in addition to extracellular dopamine diffusion and clearance.

Functionalized SWCNTs can further be applied to detect other biomolecules [183] such as oxytocin [184] and glucose [185] in brain tissue, and cellular ATP [186]. One caveat of CoPhMoRe‐based systems is that their detection specificity is dictated by the adsorbed polymer corona [183]. While this provides versatility for detecting distinct analytes, each new target analyte requires extensive polymer screening or molecular design, which may limit the scalability and rapid translation to new biomarkers. Looking ahead, advances in computational modeling, particularly in machine learning approaches, are expected to accelerate the design of surface functionalized SWCNT sensors to achieve higher biomolecule detection specificity and sensitivity [187, 188, 189].

## 5 Two‐Dimensional (2D) Materials

Beyond graphene and other 2D carbon allotropes, semiconducting transition metal‐based 2D materials such as molybdenum disulfide (MoS_2_) and MXene (transition‐metal carbides and nitrides) also exhibit strong optical responses to local field perturbations [190]. These materials further expanded the landscape of transducing cell signals into optical readouts [191, 192].

### 5.1 Bioelectric Signal Detection

Ren et al. introduced a trionic mechanism for label‐free optical recording of bioelectric signals from hiPSC‐derived cardiomyocytes leveraging the photoluminescence (PL) effect of MoS_2_ monolayers [193]. This approach harnessed the exciton‐charged exciton (trion) conversion governed by Fermi‐Dirac statistics, such that when the Fermi level shifts due to local electric fields, the PL intensity changes through increased trion formation [194]. This relationship allows voltage detection using a MoS_2_ monolayer without external biasing due to its high trionic density in the as‐grown state. Under modulated electrical stimulation, the trionic PL exhibited approximately a 20% change in fractional intensity when 1 kHz, ±10 mV squarewave potentials were applied, corresponding to a voltage detection sensitivity of ∼60 µV Hz^−^^1/2^ (Figure 13a). The optical response also follows the sign of the applied squarewave modulation (Figure 13b). When interfaced with cardiomyocytes, the MoS_2_ monolayer functions as both the optical sensor and the electrode (Figure 13c,d). Under the multimodal recording setup, the trionic PL transients collected from the MoS_2_ underneath the cell aligns well with the synchronized extracellular electrode recordings (Figure 13e).

**FIGURE 13:**
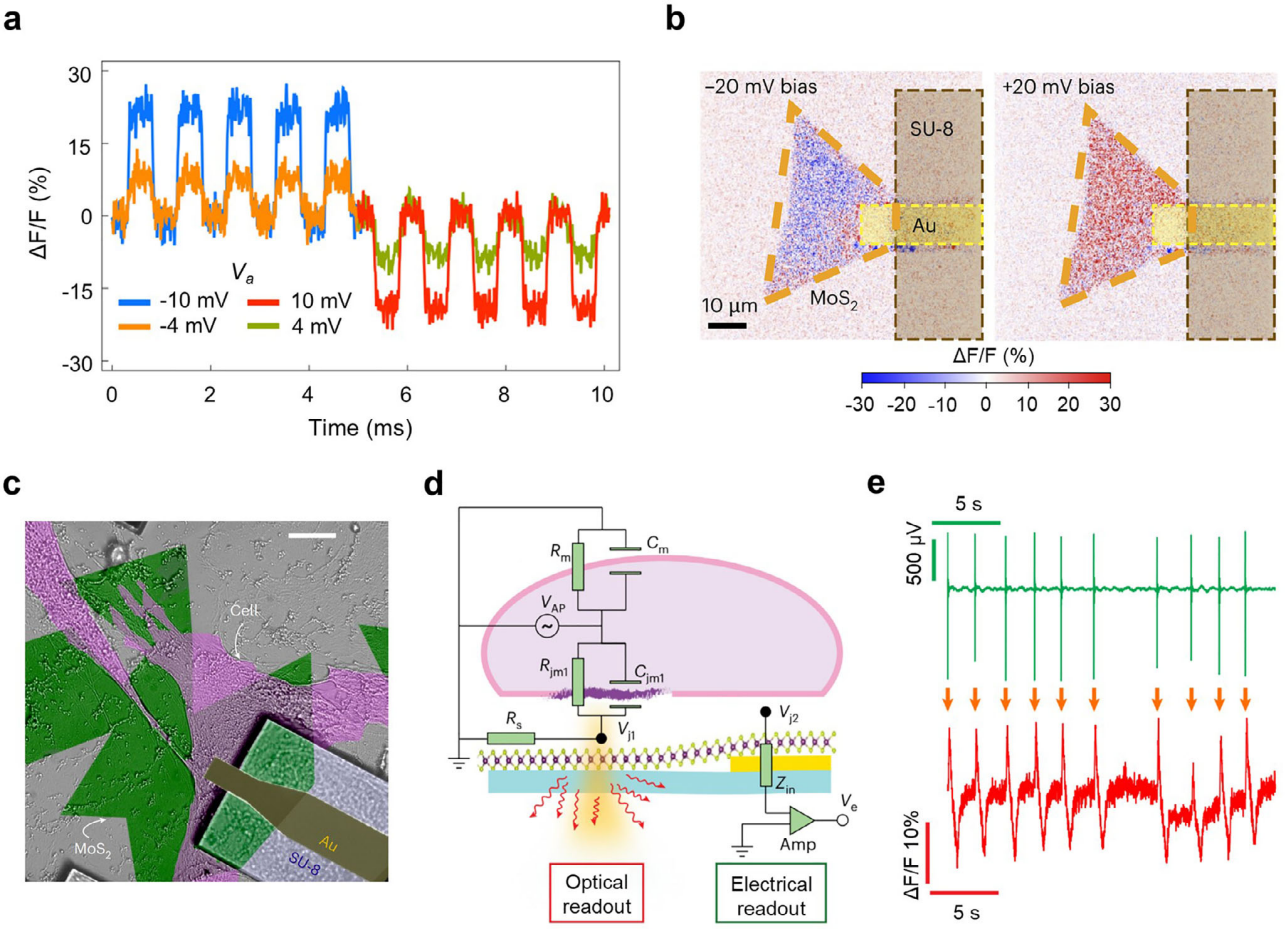
Optical recording of bioelectric signals using MoS_2_ monolayers. (a) Time‐resolved fractional PL intensity change (Δ*F*/*F*) of MoS_2_ under applied ±10 mV and ±4 mV square wave potentials at 1 kHz. (b) Fluorescence microscopy images of MoS_2_ under negative (left) and positive (right) applied voltage bias. (c) False color micrograph of the MoS_2_ multimodal recording configuration. A cardiomyocyte (purple) is on top of a MoS_2_ monolayer (green). (d) Circuitry of the multimodal detection platform, where V_j1_ and V_j2_ correspond to extracellular potentials recorded optically and electrically. (e) Time‐synchronized electrical (green) and photoluminescence (red) recording traces. Panels (a–e) reproduced with permission from Ref. [193]. Copyright 2025, Springer Nature.

Although the voltage sensitivity of this technique is currently lower than microelectrode arrays, the atomically thin MoS_2_ layer enables highly localized optical measurements with potential subcellular spatial resolution, making this technique well‐suited for mapping heterogeneous electrical activities. Furthermore, the high recording bandwidth (>50 kHz) is well above that required to record fast bioelectric signals such as neuronal extracellular action potentials. The primary limitation is the relatively low quantum efficiency, which requires high excitation intensity to maintain a sufficient recording signal‐to‐noise ratio. Further improvement in quantum efficiency through chemical or plasmonic enhancement is needed to lower the required excitation power while increasing voltage sensitivity.

Overall, this study paves the way for label‐free optical electrophysiology based on 2D materials. Besides recording bioelectric signals, MoS_2_ nanolayers and QDs have also been used for optical detection of nucleic acids and cellular imaging leveraging their strong PL effect [195, 196], as well as for therapeutic applications harnessing their high photothermal conversion efficiency [197, 198, 199]. These topics are beyond the scope of this review and we refer the readers to other comprehensive reviews [200, 201, 202].

### 5.2 Biochemical Signal Detection

MXenes can function as optical reporters of biochemical activities when engineered at the nanoscale. Wang et al. developed a chimeric DNA‐functionalized MXene probe (dcDNA‐Ti_3_C_2_) to simultaneously sense mucins and microRNAs in cancer cells [203]. In their design, a chimeric DNA construct is anchored to the MXene surface through π–π and electrostatic interactions and undergoes efficient fluorescence quenching via energy transfer (Figure 14a). Specific binding of a mucin 1 (MUC1) aptamer or hybridization of microRNA‐21 (miR‐21) induces physical separation of the fluorophore‐labeled DNA from the MXene surface, which relieves quenching and produces a spatially resolved fluorescence. The resulting signals for MUC1 and miR‐21 appear at the membrane and within the cytoplasm, respectively, and can be read out orthogonally through spectral separation of the fluorescence channels (Figure 14b). This method enabled multichannel imaging of both cancer‐associated biomarkers at nanomolar concentrations. Another aptamer‐modified MXene was developed by Guan et al. for the detection of ATP and GTP [204]. In this system, fluorophore‐labeled nucleotide aptamers were initially quenched on the MXene surface via FRET (Figure 14c). After selective binding with ATP and GTP, the aptamers desorb and fluorescence is restored to provide the optical readout. Coupled with photothermal stimulation, this sensing strategy enables real‐time, single‐cell monitoring of ATP and GTP dynamics during nucleolar stress. Similar to polymer nanoparticle FRET sensors, MXene‐based FRET sensors rely on target‐induced probe dissociation to restore fluorescence, limiting continuous monitoring of reversible biochemical dynamics. Their reliance on fluorescent labels also introduces photobleaching, variable labeling efficiency, and probe degradation, which can compromise long‐term quantitative intracellular measurements.

**FIGURE 14:**
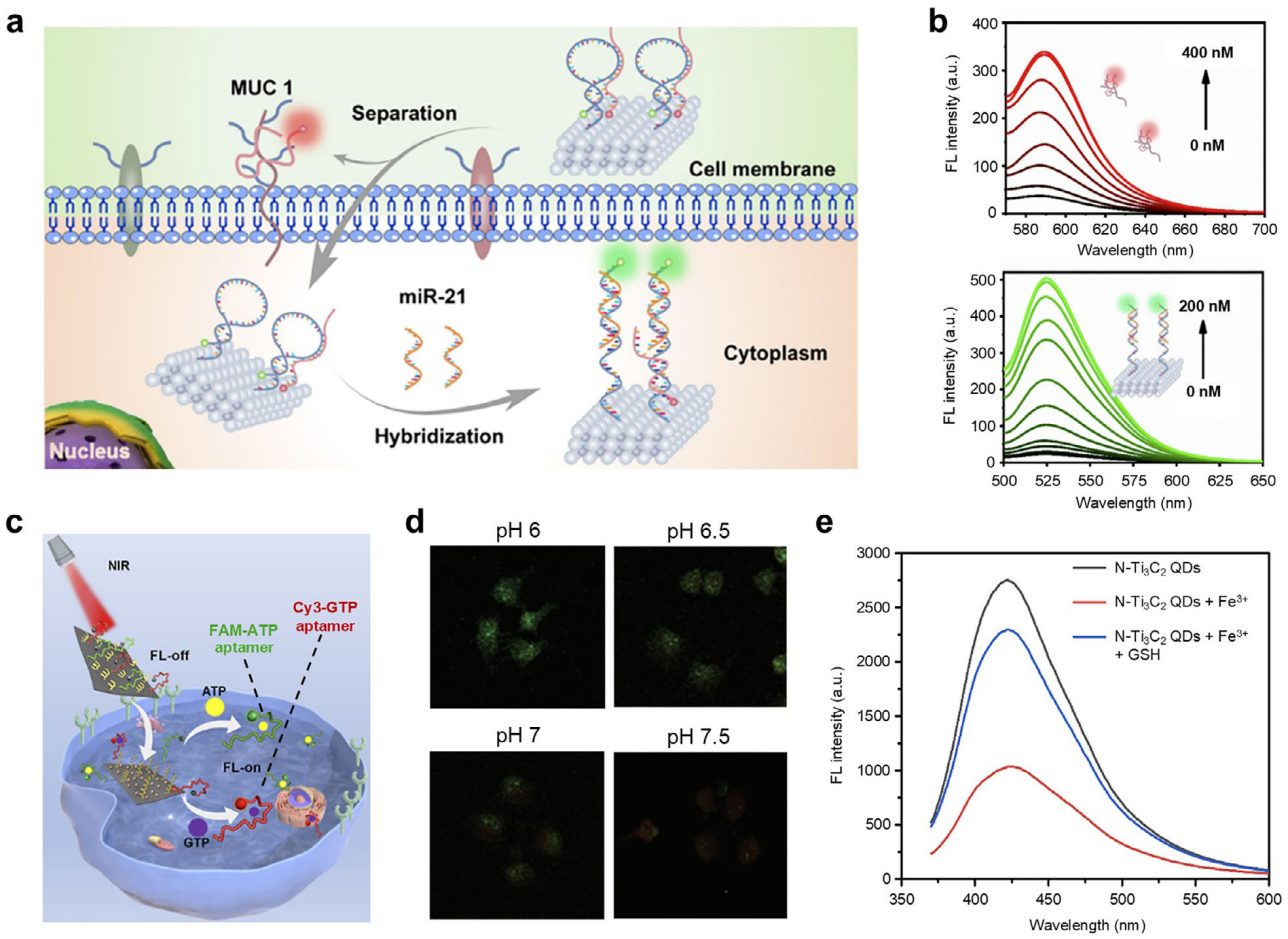
MXene‐based nanoplatforms for multiplexed optical detection of nucleic acids, nucleotides, and redox‐active biomolecules. (a) Imaging mechanism of MUC1 and miR‐21 using dcDNA‐Ti_3_C_2_. MUC1 binding to the tetramethylrhodamine‐labeled aptamer (red) induces red fluorescence at the cell membrane and miR‐21 interaction with the fluorescein amidite‐labeled DNA (blue) induces green fluorescence within the cytoplasm. (b) Fluorescence spectra of dcDNA−Ti_3_C_2_ under different concentrations of MUC1 (top) and miR‐21 (bottom). (c) Working mechanism of ATP and GTP monitoring in living cells using Ti_3_C_2_T_x_ nanoprobes functionalized with fluorescently labeled aptamers. (d) Confocal microscopy images of MCF‐7 cells at pH 6.0, 6.5, and 7.5 with MXene QDs. (e) Photoluminescence spectra of the NTi_3_C_2_ QDs excited at 350 nm under different conditions. Panels (a and b) reproduced with permission from Ref. [203]. Copyright 2019, American Chemical Society. Panel (c) reproduced with permission from Ref. [204]. Copyright 2025, American Chemical Society. Panel (d) reproduced with permission from Ref. [205]. Copyright 2018, Royal Society of Chemistry. Panel (e) reproduced with permission from Ref. [206]. Copyright 2021, Elsevier B. V.

Beyond aptamer‐based MXene sensors, MXenes have also been formulated into QDs to detect biochemical signals. Xu et al. fabricated MXene QDs that display photoluminescence with a reversible, pH‐dependent intensity change arising from protonation‐controlled surface defects [205]. When paired with the reference dye Ru(dpp)_3_^2+^, these MXene QDs enable intracellular pH tracking in live MCF‐7 cancer cells (Figure 14d). The detected photoluminescence is highly stable over repeated pH cycling with less than 5% spectral crosstalk to allow reliable intracellular pH quantification. Recently, Luo et al. employed nitrogen‐doped MXene QDs in a redox‐activated sensing scheme for intracellular glutathione detection [206]. Under basal conditions, the photoluminescence of MXene QDs remains in an “OFF” state with Fe^3+^ ions acting as quenchers. When exposed to elevated intracellular glutathione levels in cancer cells, Fe^3+^ is reduced to Fe^2+^, thereby suppressing quenching and turning on the QD photoluminescence (Figure 14e). This detection method achieved a detection sensitivity of ∼0.17 µM. The probe also showed good biocompatibility, with over 90% cell viability after 24 h of incubation at concentrations up to 200 µg mL^−^^1^.

Although MXenes exhibit promising biocompatibility under many experimental conditions, their cytotoxicity remains an active area of investigation [207, 208]. Nevertheless, these concerns have not hindered research progress. Instead, these motivated studies aimed at leveraging the exceptional optical properties of MXenes for cell signal detection and at the same time ensuring safety [209].

## 6 Quantum Materials

### 6.1 Diamond With Nitrogen Vacancy (NV) Color Centers

Diamond crystals hosting nitrogen‐vacancy (NV) defects represent a solid‐state quantum material capable of storing and manipulating spin states (qubits) with exceptional coherence [213]. When a negatively‐charged NV center is excited with green light (532 nm), it emits red fluorescence with an intensity that depends on the strength of the local magnetic fields [214]. Due to the electronic structure of NV centers, excited electrons in the spin state (*m*_s_) = ±1 are more likely to undergo non‐radiative decay than those in the *m*_s_ = 0 state [215]. In consequence, when microwaves at the resonance frequency of the *m*_s_ = 0 → ±1 transition are applied, the resulting fluorescence signal sees two dips corresponding to the decay of excited *m*_s_ = ±1 electrons to ground states (Figure 15a). The spacing between these dips reflects how the energy levels split in response to a magnetic field. By measuring this separation, the local magnetic field at the NV center can be quantified [216]. This principle, first demonstrated by Jelezko et al., is known as optically detected magnetic resonance (ODMR) [217]. ODMR provides a pathway for optically detecting transient magnetic signatures associated with cell electrical activity and, in related NV‐based implementations, paramagnetic chemical processes.

**FIGURE 15:**
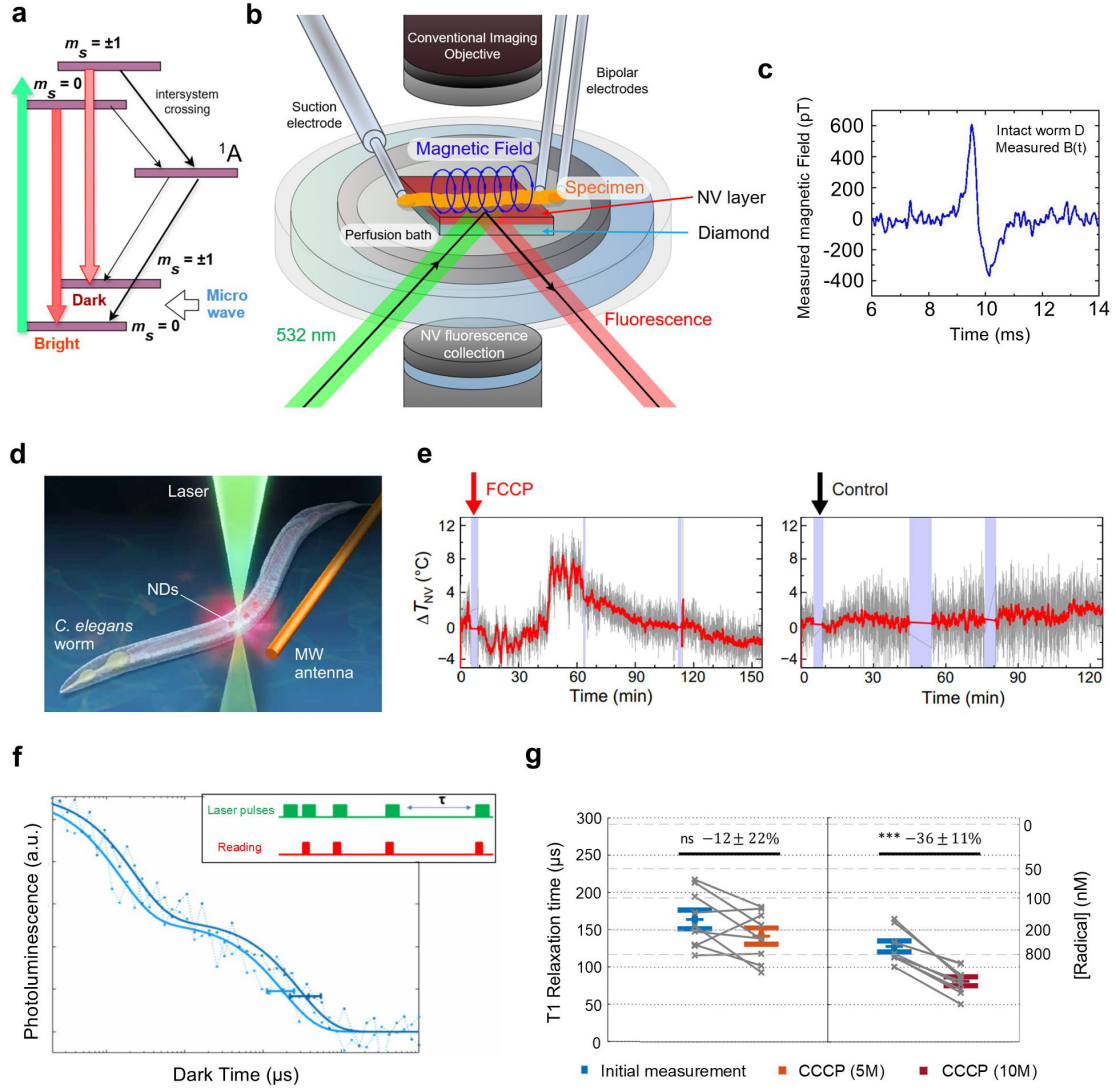
Bioelectric and biochemical signal detection using nitrogen‐vacancy (NV) center in diamond. (a) Energy level diagram of a diamond NV center. Optical excitation (green) promotes population to the triplet excited state (^3^E) followed by spin‐dependent radiative decay (red). Microwave excitation drives transitions between spin sublevels that enable optical readouts. (b) Setup schematic for detecting magnetic fields accompanied by neuroelectric signals using NV diamond. The biological specimen (orange), with its associated magnetic field (blue), is positioned on the NV‐active surface of a diamond chip. NV centers are excited with a 532 laser, and the resulting fluorescence is collected. Electric stimuli are applied via the suction electrode and are monitored using bipolar electrodes. (c) Time‐resolved magnetic field trace of action potentials in *Myxicola infundibulum* averaged over 600 events. (d) Schematic of NV diamond thermometry setup. (e) Measured temperature change with FCCP stimulation (left) and control (right). (f) Time trace of photoluminescence from control nanodiamonds (dark blue) and VDAC2 antibody‐coated nanodiamonds (light blue). Inset: pulsing sequence of laser excitation (green) and photoluminescence collection (red). (g) Free radical concentration measured by T_1_ relaxation time of nanodiamonds comparing control (blue) with 5 nM (orange) and 10 nM (red) CCCP‐treated mitochondria. Panels (b and c) adapted from Ref. [210]. Panels (d and e) adapted from Ref. [211] under the CC BY 4.0 license. Copyright 2020, The American Association for the Advancement of Science. Panels (f and g) adapted from Ref. [212] under the CC BY‐NC 4.0 license. Copyright 2021, The American Association for the Advancement of Science.

#### 6.1.1 Bioelectric Signal Detection

Bioelectric activity inherently generates magnetic fields. Using NV diamond ODMR, Barry et al. recorded the extracellular action potentials from the worm *Myxicola infundibulum* and giant squid axons by detecting the accompanied magnetic fields [210]. A uniform, high‐density layer of NV centers embedded at the top surface of the single‐crystal diamond (∼3 × 10^17^ cm^−3^) is positioned in direct contact with the biological specimen (Figure 15b). The magnetic fields associated with the action potential generated by the specimen interact with the NV centers. As microwaves are applied, a photodiode collects the fluorescence signal for ODMR readout. Using continuous‐wave electron spin resonance magnetometry, time‐dependent changes in dip separation in the ODMR spectrum are measured and converted into magnetic flux density. Because the dip separation reverses sign depending on the magnetic field projection at the NV center, this method can also determine the direction of action potential propagation (Figure 15c). The measurement achieves an SNR of ∼1.2 for a single action potential firing and a magnetic field sensitivity of ∼10 pT Hz^−½^, which is remarkable given the faint responses in the pT–nT range. However, these values represent the ensemble average over many recording data traces. To record action potential from single mammalian cells, optimizing the magnetic field detection SNR is needed [218, 219].

#### 6.1.2 Biochemical Signal Detection

Measuring heat released from cells can provide valuable insight into cell chemical processes. Thermal fluctuations can serve as an indirect probe of cell energy metabolism. Processes such as oxidative phosphorylation, including ATP synthesis, dissipate a fraction of their energy as heat [220]. Fujiwara et al. used NV diamond ODMR to detect thermogenic activity in the worm *Caenorhabditis elegans* [211]. The authors adopted a similar approach to Barry et al., but the ODMR dip frequency shifts are driven by temperature changes rather than magnetic fields. The thermal expansion of the nanodiamond lattice induces crystal strain and distorts defect symmetry, thereby shifting the energy of ground‐state electron spin sublevels [221]. The NV nanodiamond‐labeled specimen is placed on a microwave antenna‐integrated glass dish and illuminated with a green laser (Figure 15d). ODMR spectra are recorded and the total photon counts are converted into temperature estimates (Δ*T*_NV_) as a function of time (Figure 15e, *left*). An increase in Δ*T*_NV_ is detected when thermogenesis is induced by carbonyl cyanide *p*‐trifluoromethoxyphenylhydrazone (FCCP) through uncoupling of oxidative phosphorylation (Figure 15e, *right*). The recording has a temporal resolution of ∼1 s. Although data averaging was still required, NV nanodiamond thermometry overcomes the bulkiness and limited spatiotemporal resolution of conventional thermometers and is suitable for long‐term monitoring of cell temperature fluctuations [222]. Nonetheless, cell thermogenesis remains an epicenter of debate because extracellular temperature measurements often appear inconsistent with the theoretical maximum cellular heat output [223]. Clarifying the origins of such inconsistencies will be important for optimizing thermometry as a reliable biosensing tool.

Besides ODMR, NV diamond can probe cell reactive oxygen species (ROS) through relaxometry. This method leverages the sensitivity of NV spin relaxation times (*T*_1_) to magnetic noise from unpaired electron spins to provide a measure of free radical concentration [224]. In a study by Nie et al., NV nanodiamonds functionalized with antibodies were internalized into J774A.1 macrophages (mouse immune cells) [212]. A pulsed laser sequence with variable dark times was used to excite the NV nanodiamonds and the fluorescence intensity was measured to extract the relaxation time *T*_1_ (Figure 15f). Carbonyl cyanide *m*‐chlorophenyl hydrazone (CCCP) was then added to the cell to increase ROS formation in the mitochondria, which produced a detectable shift in the relaxation curve and a shortened *T*_1_ (Figure 15g). Since the same nanodiamond can be repeatedly measured within an individual cell over time, this approach distinguishes dynamic metabolic changes from cell‐to‐cell variability, providing a key advantage for longitudinal monitoring of mitochondrial function. *T*_1_ relaxometry has also been used to detect free radicals in other biological systems such as mouse liver slices [225], human dendritic cells, and breast cancer cells [226]. Despite its advantage, *T*_1_ relaxometry remains technically demanding, requiring specialized quantum sensing instrumentation and relatively long acquisition times (∼2 min) to achieve sufficient sensitivity. These requirements may limit its applicability to detect rapid biochemical processes.

## 7 Metals

### 7.1 Metal Thin Films With Non‐Plasmonic Methods

#### 7.1.1 Biomechanical Signal Detection

##### 7.1.1.1 Optical Cavity

An optical cavity uses two or more reflective interfaces to confine light through repeated internal reflections to produce constructive interference at discrete resonant frequencies. Low optical loss yields a high quality factor (*Q*) and is characterized by narrow resonance peaks and long photon lifetimes. Mechanical force or strain altering the cavity length or spacer refractive index can shift the optical path length and resonance frequency. Measuring these shifts therefore enables highly sensitive detection of biomechanical signals.

Gather group developed an elastic resonator interference stress microscopy (ERISM) method where cells are cultured on an elastic microcavity composed of two semitransparent gold mirrors laminating an elastomer [227]. Cell‐force‐induced displacement produces interferometric changes in the cavity and shifts the resonant interference. The nanometer‐scale displacement is then converted to stress using finite‐element modeling. ERISM allows long‐term, low‐phototoxic imaging and provides piconewton‐scale force resolution with axial displacement sensitivity on the order of ∼2 nm [228]. Wavelength‐alternating resonant pressure microscopy (WARP) was further developed by replacing slow wavelength scanning in ERISM with two rapidly alternating wavelengths to achieve high‐speed interferometric force imaging (Figure 16a) [229]. WARP enhances temporal resolution to 100 frames per second (fps) and enables real‐time displacement mapping of the spontaneous beating and contraction of neonatal mice cardiomyocytes with nanometer resolution (Figure 16b,c) [229]. Recently, the authors extended WARP to quantify ground‐reaction forces in *Drosophila* larvae on the organism level [230]. De Angelis group employed a similarly interferometric microcavity approach using a thin membrane embedded with gold micromirrors (Figure 16d) [231]. Different from ERISM or WARP, this approach fills the cavity with a fluorescent dye instead of an elastomer. During cardiomyocyte contraction, membrane deformations alter the cavity thickness and shift the interference phase, consequently modulating the fluorescence intensity (Figure 16e). This approach resolves displacement on the order of ∼40 nm with an SNR of ∼10 and enables traction‐stress estimates of ∼34 ± 4 mN/mm^2^ (Figure 16f).

**FIGURE 16:**
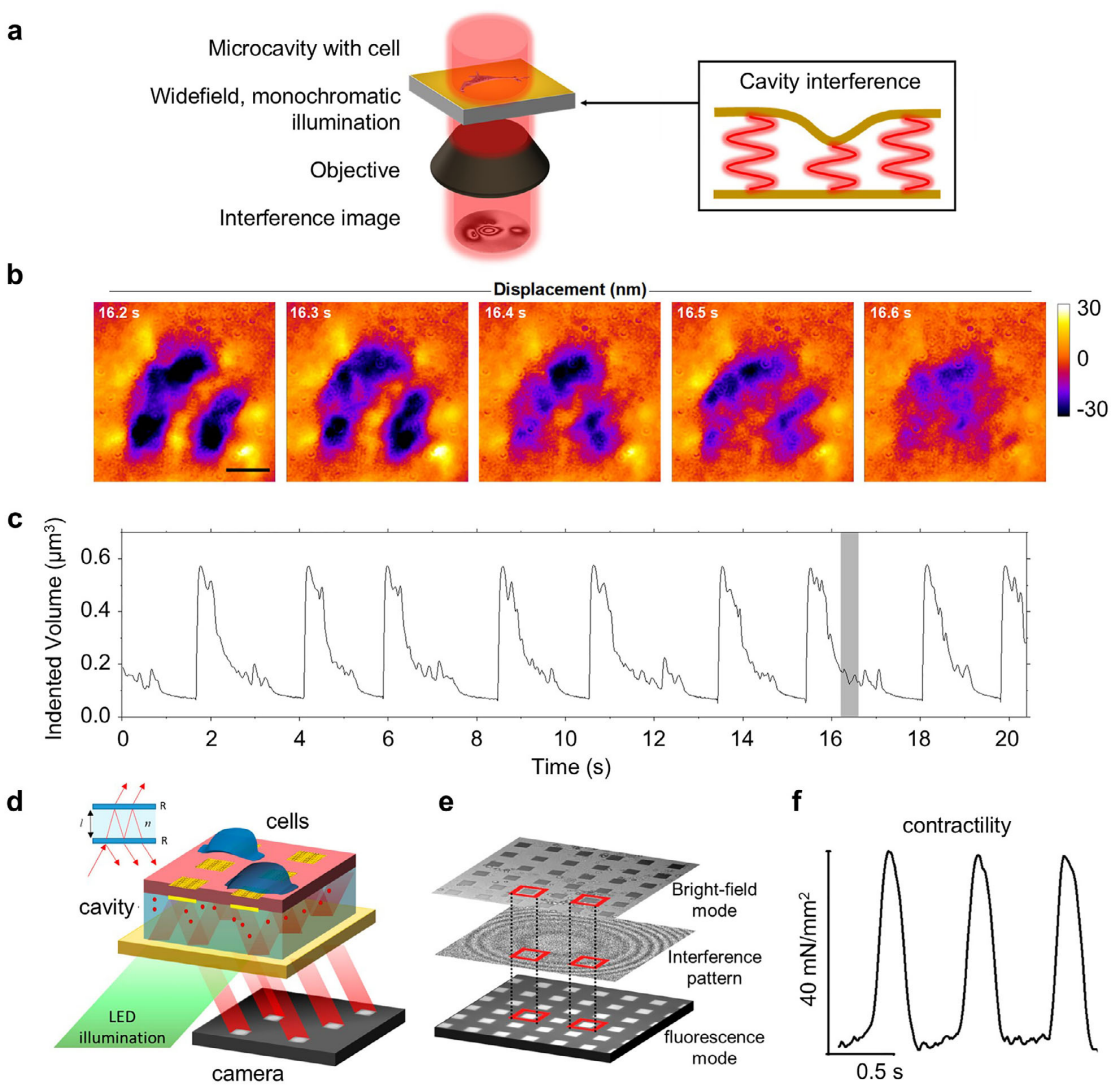
Biomechanical signal detection using metal optical cavity. (a) Schematic of WARP setup. Cells are cultured on the top mirror of an elastic microcavity chip. Cell‐generated forces locally deform the cavity, change its thickness, and produce an interference fringe pattern. (b) WARP imaging of cardiomyocytes at 100 fps shows characteristic contractions. (c) Contraction time trace of cardiomyocytes over nine contraction cycles. The grey box indicates the specific period in (b). (d) Schematic of the device with a silicon nitride membrane (red) embedded with gold mirrors. The cavity is filled with ethylene glycol with 0.2 mg/mL rhodamine. Gold mirrors reflect the fluorescence to be captured by a CMOS camera. (e) Schematic of image acquisition process that captures the interference pattern. (f) Example trace of three contraction events from a single microinterferometer. Panels (a–c) reproduced from Ref. [229] under the CC BY 4.0 license. Copyright 2021, The Author(s). Panels (d–f) reproduced with permission from Ref. [231] under the CC BY 4.0 license. Copyright 2024, American Chemical Society.

##### 7.1.1.2 Diffraction‐Based Method

Lin et al. reported a label‐free optical mapping method that converts cell‐induced lateral displacements of a PDMS membrane laminated between two complementary metal layers into diffraction signals [232]. Each micro‐element combines a top metal disk (on PDMS, mobile) and a bottom metal sheet with a central aperture (on glass, fixed), to form an “eclipse‐like” geometry. Illumination is largely blocked and when cell traction shifts the top disk relative to the aperture, it will dynamically modulate the transmitted and edge‐diffracted light. In cardiomyocyte monolayers, the system achieves wide‐field imaging (10.6 × 10.6 mm^2^) with 20 µm spatial and 130 fps temporal resolution. The imaging SNR is sufficient to resolve the beating frequencies and local beating heterogeneities from 2.3 Hz to 2.7 Hz. This approach supports over 7 days of label‐free beating monitoring, and can be combined with calcium imaging. It offers stronger optical modulation than grating‐only PDMS substrates (discussed in Section 2.2.1.B) due to the dual‐metal architecture. However, such architecture requires uniform PDMS thickness and demands precise fabrication. Stable light illumination and a high‐speed imaging camera are also required.

### 7.2 Metal Thin Films for Surface Plasmon Resonance (SPR)

Surface plasmon resonance (SPR) couples *p*‐polarized light into a surface‐plasmon polariton at a metal interface that produces a sharp reflectance dip at a specific angle or wavelength. In biosensing, a widely used SPR configuration is the Kretschmann geometry (thin‐film‐on‐prism, typically 40–50 nm gold film on glass) [233]. Perturbations occurring within the evanescent field (typically ∼100–300 nm in penetration depth) alter the resonance condition, which can be read out as angular, spectral, or intensity shifts [234]. SPR is well‐suited for label‐free optical interrogation of cellular processes at the cell‐material interface because its sensing volume is confined to the near‐surface region [235].

#### 7.2.1 Bioelectric Signal Detection

Kim et al. established an early proof‐of‐concept for label‐free optical recording of neural activities. Using a low‐noise Kretschmann SPR platform, they showed that electrically‐evoked compound action potentials in rat sciatic nerve generate time‐locked SPR transients that co‐vary with the simultaneous electrode‐recorded trace [236]. This approach was then translated to in vivo settings using a fiber‐based SPR probe to monitor forepaw‐evoked activity in the somatosensory cortex of anesthetized rats [237]. Tao and coworkers reframed SPR as a pixel‐resolved imager named plasmonic electrochemical impedance microscopy (P‐EIM). P‐EIM converts local voltage‐dependent impedance at the metal–electrolyte interface into an intensity map [238] and allows label‐free imaging of action potential initiation and propagation in cultured neurons (Figure 17a) [239]. The optical signals were validated through simultaneous patch‐clamp recordings and pharmacological testing with tetrodotoxin (TTX). However, later publications by the authors indicate that these SPR signals could arise from the action‐potential‐induced mechanical deformations in cell membranes [240, 241]. More recently, the authors applied matched filtering and deep learning to P‐EIM time series to advance this method towards real‐time action‐potential detection in a single shot without extensive data averaging (Figure 17b) [242].

**FIGURE 17:**
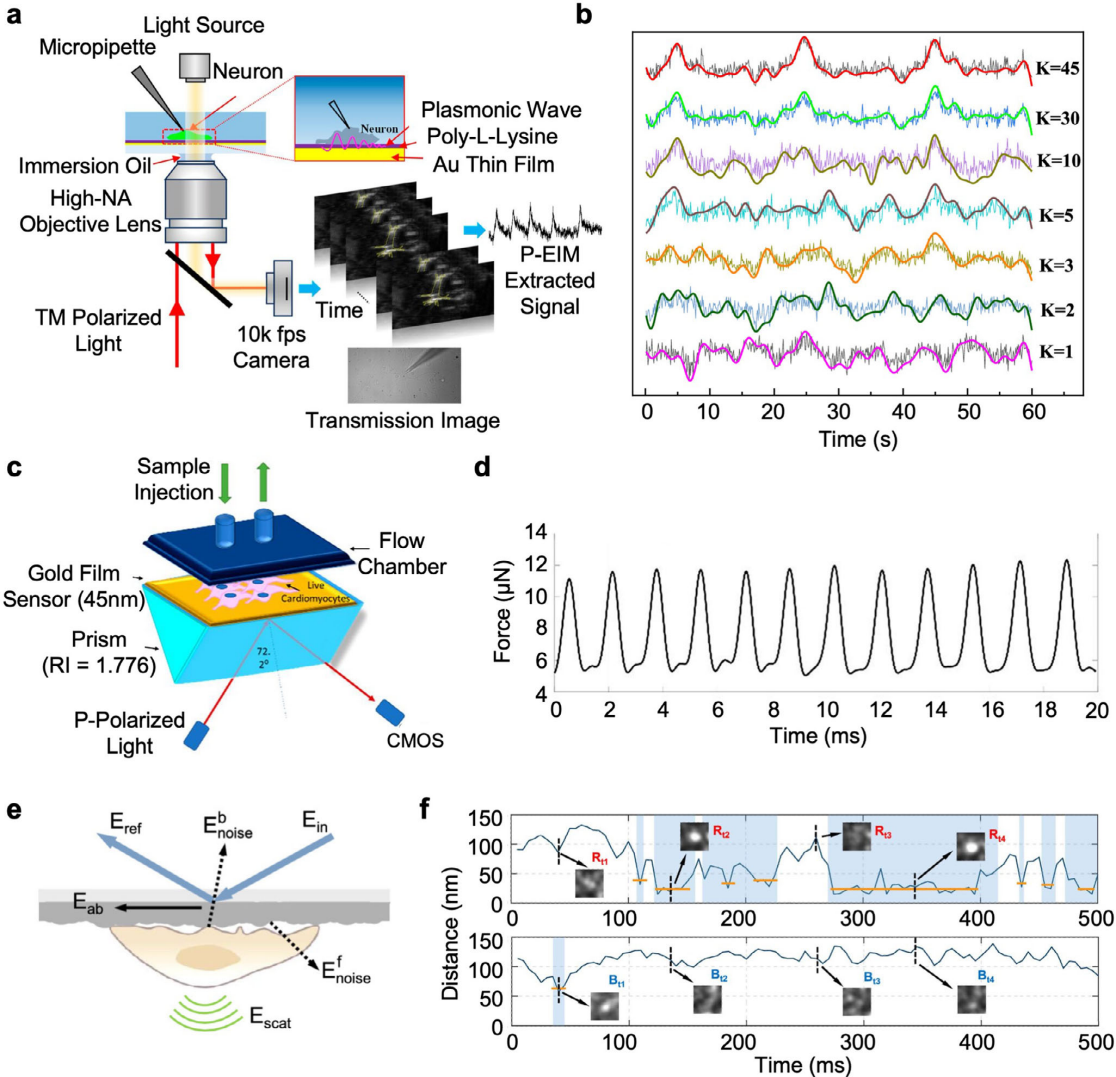
Optical detection of biomechanical signals using surface plasmon resonance. (a) Schematic of the plasmonic electrical impedance microscopy (P‐EIM) system. Surface plasmons are generated using a high‐numerical‐aperture objective on a gold‐coated glass coverslip. Images are recorded by a high‐speed camera at 10 000 frames per second. Neurons cultured on the chip are stimulated using a patch‐clamp. (b) Averaged plasmonic recording signals of action potentials using an infinite impulse response (IIR) Chebyshev low‐pass filter with different iterations (K). (c) Schematic of a cell‐embedded SPR sensor on a liquid flow system. P‐polarized light is directed at the surface of the gold layer and monitored using a CMOS camera. (d) Force profile over time of cardiomyocytes cultured on a sample chip. (e) Schematic of the PSM. The total incident light energy, *E*_in_, can be converted into the reflected light energy (*E*_ref_), the scattering signal from the sample (*E*_scat_), the light absorbed by the silver (*E*_ab_), and the backward (*E*_noise_^b^) and forward scattering (*E*_noise_^f^) due to the roughness of the metallic surface. (f) Real‐time dynamic traces for two regions on a single cell. The blue vertical bands indicate adhesion site formation. The yellow lines indicate adhesion stabilization. Panels (a and b) adapted with permission from Ref. [242]. Copyright 2024, American Chemical Society. Panels (c and d) adapted with permission from Ref. [15]. Copyright 2019, American Chemical Society. Panels (e and f) adapted with permission from Ref. [244]. Copyright 2025, American Chemical Society.

We note in the work discussed above, SPR does not measure voltage directly. Instead, it detects changes in the dielectric environment (refractive index) within the evanescent field near the gold surface. Hence, rather than a direct optical readout of the transmembrane action potential, the SPR signal is more rigorously described as an optical correlate of the action potential. These signals can arise from a combination of interfacial charge redistribution and membrane displacement (e.g., swelling) [243].

#### 7.2.2 Biomechanical Signal Detection

SPR has also allowed robust biomechanical signal detection. Giebel et al. first used SPR imaging (SPRi) leveraging an aluminum‐coated glass prism to visualize cell‐substrate contacts [235]. The near‐field evanescent wave converts nanometer changes in contact distance between the cell membrane and the substrate into a label‐free optical readout. Peterson and coworkers used wide‐field prism‐coupled SPRi to track live vascular smooth muscle cells cultured on fibronectin [245] and how cell–substrate interactions evolve over time [246]. The authors then employed a high‐numerical‐aperture objective to transform prism‐coupled SPRi into a microscopy platform and achieved diffraction‐limited spatial resolution [247]. Kim et al. further introduced SPR imaging ellipsometry (SPRIE). By incorporating phase and polarization contrast, SPRIE improves detection sensitivity to subtle mechanical deformations at the cell–substrate interface compared with intensity‐only SPRi [248]. Mozneb et al. employed prism‐coupled SPR with a microfluidic flow chamber to monitor the spontaneous beating of neonatal rat cardiomyocytes and modulated contractility using blebbistatin (inhibition) and ATP (enhancement) (Figure 17c,d) [15]. Compared with earlier studies focused mainly on cell adhesion or morphology, this work extends SPR to measure functional contractility in living excitable cells and provides guidance for its use in drug screening.

Beyond 2D cell interfaces, Zhao group integrated digital holography with SPR to develop surface plasmon holographic microscopy (SPHM) for 3D cell interfaces [249]. By incorporating wavelength multiplexing and a common‐path configuration, SPHM enables simultaneous amplitude and phase SPR imaging over a large field of view [250]. This allows mapping of live‐cell dynamics and separation of distance‐dependent effects from refractive‐index changes at the cell membrane [251, 252]. In contrast to SPHM that reconstructs amplitude and phase to provide depth‐resolved maps of adhesion‐gap thickness, You et al. developed a plasmonic scattering microscopy (PSM) that enables robust axial localization and multisite 3D mapping of cell adhesion and traction dynamics by exploiting the plasmonic scattering pathway (Figure 17e) [253]. Rather than recovering the absolute phase, PSM infers axial position from calibrated scattering signals that trade phase quantification for speed and simplicity while preserving near‐field sensitivity. Therefore, PSM supports real‐time, label‐free 3D mapping of live cell‐substrate adhesion by adding a depth‐resolved profiling (Figure 17f) [244].

### 7.3 Nanostructures for Localized Surface Plasmon Resonance (LSPR)

Besides continuous metal thin films, fabricated periodic nanostructures can also be leveraged for localized surface plasmon resonance (LSPR) [254]. LSPR can produce very sharp resonance peaks with high sensitivity (high figure of merit). Nanostructures such as gold nanoshells and nanorods also allow tuning the resonance peaks into the near‐infrared (NIR) region [255, 256].

#### 7.3.1 Bioelectric Signal Detection

Nurmikko group shows that a planar gold nanoparticle (AuNP) array attached to mammalian neurons can act as local electrostatic field sensors. By monitoring time‐resolved LSPR scattering intensity and spectral shifts from single nanoparticles near the cell membrane, the authors correlate spontaneous and stimulated neuronal activities with optical readouts [257]. Yanik group demonstrated electro‐plasmonic nanoantennas (Figure 18a,b) built from lithographically patterned gold nanoantennas coated with an ultrathin electrochromic PEDOT:PSS layer on an ITO substrate. This design enables LSPR detection of field changes induced by cardiomyocytes cultured on nanoantennas through changes in light scattering intensities with a sub‐millisecond (<0.2 ms) temporal response (Figure 18c) [258]. Recently, Sarkar group introduced organic electro‐scattering antennas (OCEANs), which are micrometer‐scale PEDOT:PSS “mushroom” shape protrusions on an ITO substrate that convert local voltage‐driven doping/dedoping of the polymer into visible‐light scattering changes. This approach enables optical potential readout with a ∼2.5 mV sensitivity and ∼5 µm spatial resolution while maintaining millisecond‐scale temporal resolution [259].

**FIGURE 18:**
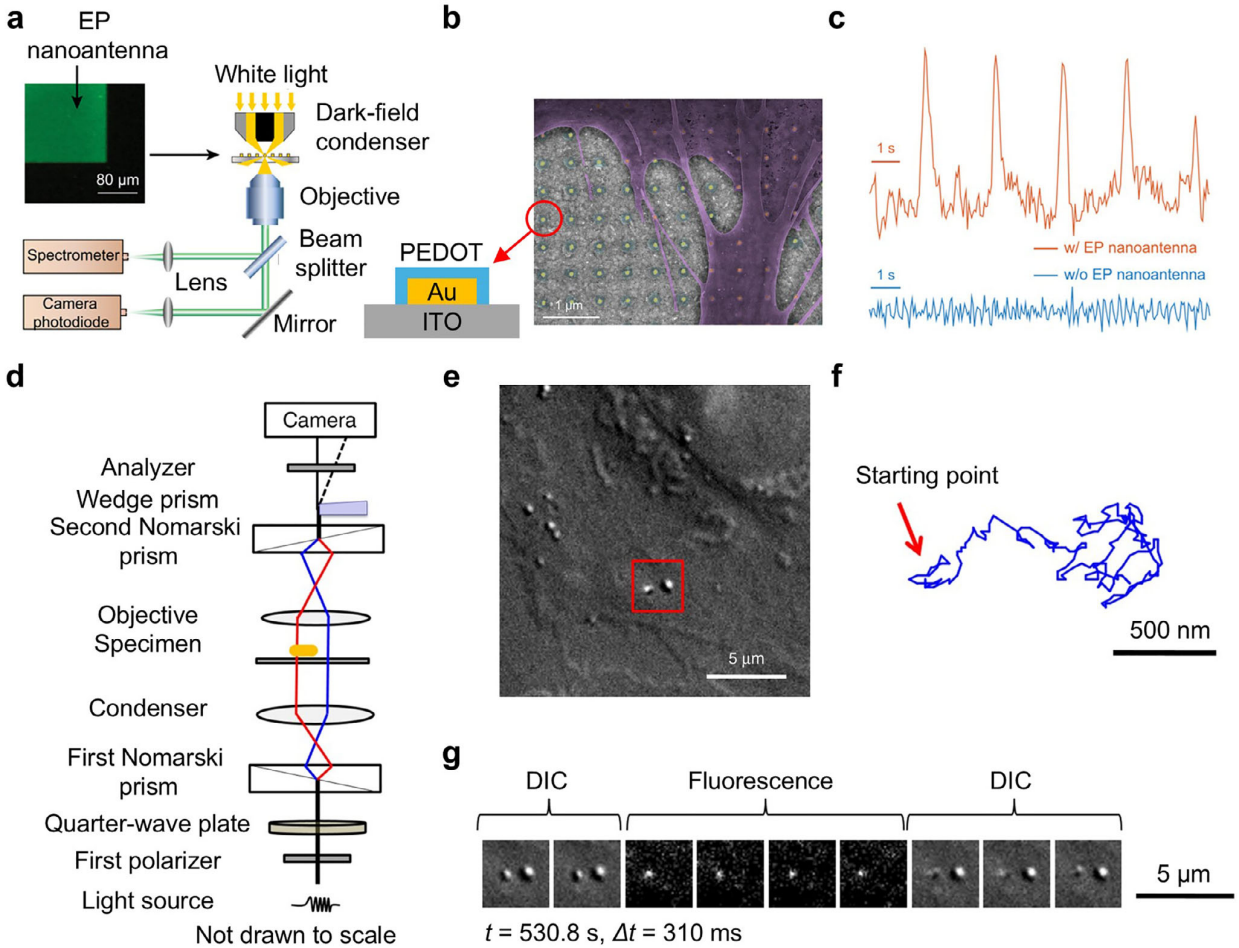
Optical detection of bioelectric and biomechanical signals using metal nanostructures. (a) Schematic of the transmission dark‐field measurement setup. Strong light scattering contrast in between the spatial regions with (green) and without (dark) electro‐plasmonic (EP) nanoantenna is observed (inset). (b) False‐color scanning electron micrograph of hiPSC‐derived cardiomyocytes (purple) cultured on an electro‐plasmonic nanoantenna array. (c) Nanoantenna scattering signal in response to the electrical activities of cardiomyocytes (orange curve). No signal is detected in the absence of electro‐plasmonic nanoantennas (blue curve). (d) Schematic of parallax‐DIC microscope. (e) A gold nanorod probe on the cell membrane. The nanorod (on the left in the red box) next to a cell feature (on the right in the red box) showed a constant contrast throughout the recording. Scale bar = 5 µm. (f) Particle tracking trace of the nanorod's active translational movement at an early binding stage. (g) Sequential DIC and fluorescence images captured in tandem shows that the gold nanorod co‐localized with a clathrin‐coated pit (CCP). Panels (a–c) reproduced from Ref. [258] under the CC BY‐NC 4.0 license. Copyright 2019, The American Association for the Advancement of Science. Panels (d–g) reproduced from Ref. [260] under the CC BY 4.0 license. Copyright 2017, The Author(s).

#### 7.3.2 Biomechanical Signal Detection

Fang group used gold nanorods (AuNRs) as orientation and rotation reporters in live cells. They combined differential interference contrast (DIC)‐based (Figure 18d) single‐particle orientation and rotational tracking (SPORT) with AuNRs to resolve rotational motions and torques of nano‐cargos during intracellular transport [261, 262, 263, 264]. This series of work links time‐resolved orientation to motor forces and binding constraints. Using membrane‐tethered nanorods, they further distinguish in‐plane rotation and out‐of‐plane tilting on the plasma membrane (5 ms temporal resolution) to read out membrane viscoelasticity, receptor‐ligand geometry, and binding strength [265]. The authors then introduced parallax DIC microscopy to simultaneously resolve the three spatial coordinates and two orientation angles of a single nanorod with ∼1 ms temporal resolution, revealing mechanochemical checkpoints during clathrin‐mediated endocytosis (Figure 18e–g) [260].

Leveraging AuNR orientation as a mechanical observable, Crocker group used a membrane‐tethered AuNR as a local reporter of membrane bending mechanics, where orientation‐sensitive dark‐field scattering tracks rotational fluctuations of the AuNR with ∼1° precision [266]. The rotation optical signal is then interpreted with theory and simulation to extract contributions from viscosity, bending rigidity, tension, and inter‐leaflet friction. Building on this concept, the Zhang group quantified the local viscoelastic moduli of macrophage lysosomes by autocorrelation analysis of dark‐field polarization intensity fluctuations caused by out‐of‐plane microrotations. They further showed that lysosomal viscosity can serve as a robust indicator of macrophage pathological states [267].

AuNRs provide bright, label‐free scattering signals that remain stable during long‐term live‐cell imaging. Because their anisotropic plasmonic response is highly orientation‐dependent, even small changes in azimuth or tilt generate distinct optical signatures that can be resolved using DIC‐based techniques, including SPORT, parallax‐DIC, and 3D orientation imaging [261]. These approaches enable tracking of biomechanical signals with high temporal resolution [260]. With appropriate calibration and physical modeling, nanorod rotational dynamics can further be used to measure viscosity or viscoelastic moduli. However, although nanorods do not photobleach, high illumination power can induce local heating. Careful power and exposure control are thus important to minimize photothermal effects [261].

#### 7.3.3 Biochemical Signal Detection

Raphael group used gold nanostructure arrays as pixelated transducers to perform wide‐field LSPR imaging near hybridoma cells to detect biochemical signals in real‐time [254]. When proteins secreted by cells bind to capture antibodies patterned on the nanostructure arrays, they alter the local refractive index and shift the LSPR resonance. The authors then resolved both burst‐like and continuous protein secretion from individual hybridoma cells and mapped pericellular secretion plumes with 10 µm spatial and 225 ms temporal resolution.

Altug group used extraordinary optical transmission (EOT) with periodic gold nanohole arrays integrated with microwells to seat single cells directly above the plasmonic transducer. Binding of secreted cytokines to antibody‐functionalized holes produces spectral shifts in EOT that are read out in a label‐free manner (Figure 19a). This method allows stable, 10‐h monitoring with minimal well‐to‐well cross‐talk [268]. Because the EOT optical readout can be easily calibrated and scaled to a multiwell format, the authors then used this approach to simultaneously track the secretion kinetics of hundreds of single cells in ∼0.4 nL compartments (Figure 19b) [269]. Machine‐learning‐assisted segmentation/tracking tools can further be applied to suppress motion‐induced artifacts in long time‐lapse recordings [270]. In addition to real‐time biochemical signal monitoring and mapping, microwell selection with SPR imaging has also been used for single‐cell capture and isolation [271] as well as detecting disease biomarkers from clinical biofluids containing exosomes [272, 273].

**FIGURE 19:**
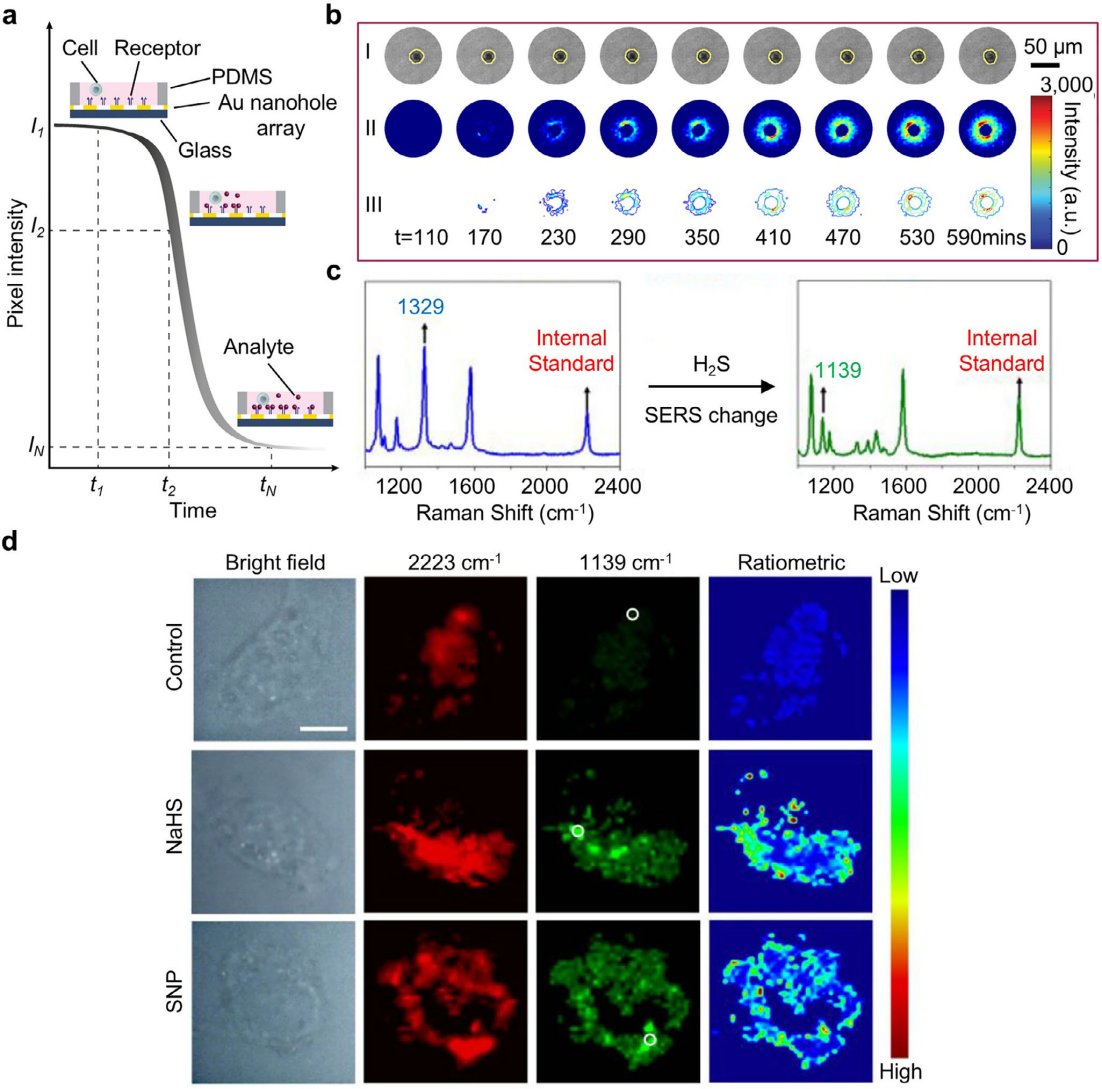
Optical detection of biochemical signals using metal nanostructures. (a) Schematic of plasmonic single‐cell microwell array. A binding event sensogram at each camera pixel is obtained by measuring the intensity changes in real time. (b) High‐efficiency light transmission through the patterned gold surface provides high‐contrast optical images that enable simultaneous analysis of secretion and morphology of the cell to establish cell secretion profiles. (c) 4‐NTP functionalized AuNP SERS probes for H_2_S detection. H_2_S reduces 4‐NTP (1329 cm^−1^) to 4‐ATP followed by dimerization to DMAB (1139 cm^−1^). MPBN (2223 cm^−1^) serves as an internal standard for ratiometric readout. (d) Confocal ratiometric SERS imaging of the exogenous and endogenous H_2_S in HepG2 cells treated with NaHS and sodium nitroprusside (SNP) using reaction‐based AuNP SERS probes. Panels (a and b) reproduced from Ref. [270] under the CC BY 4.0 license. Copyright 2023, The Author(s). Panels (c and d) adapted with permission from Ref. [274]. Copyright 2022, American Chemical Society.

### 7.4 Nanostructures for Raman‐Based Method

Surface‐enhanced Raman scattering/spectroscopy (SERS) is a vibrational spectroscopic technique that significantly amplifies the weak Raman signals of molecules by coupling them to localized surface plasmon resonances on nanostructured metals [275]. SERS substrates are commonly metals such as gold, silver, or copper in the form of colloidal nanoparticles, nanogaps, nanoantennas, or rough metal films. Recently, hybrid materials incorporating semiconducting oxides, graphene, MXenes, or polymers with metal are explored to improve the stability, flexibility, and biocompatibility of SERS substrates [276, 277]. SERS detects inelastically scattered photons generated by plasmonic excitation at a metal‐dielectric interface to produce a full Raman spectrum with molecular specificity [278, 279]. By contrast, SPR (discussed in Sections 7.2 and 7.3), monitors changes in propagating surface plasmon modes as the local refractive index changes with no molecular information [280].

#### 7.4.1 Bioelectric Signal Detection

SERS has been explored for detecting membrane potentials. Lim group developed triphenylphosphine‐modified Au nanoparticles that selectively accumulate in mitochondria and generate Raman spectra dominated by cytochrome *c* and other mitochondrial components. Changes in these spectral bands report mitochondrial membrane depolarization in real time in rheumatoid‐arthritis synovial fibroblasts under tumor necrosis factor alpha (TNF‐α), chemical, and light stimulation. Time‐resolved Raman measurements with exposure times as short as 30 ms demonstrate the feasibility of using Raman readout for tracking dynamic membrane depolarization in live cells [281].

#### 7.4.2 Biochemical Signal Detection

Engineered nanoprobes composed of small Raman‐active molecules immobilized on gold or silver nanoparticles and functionalized with targeting motifs can serve as SERS tags. When these tags localize to specific organelles or membranes, their vibrational bands shift or change in intensity upon binding to or reacting with biochemical targets [282]. This approach enables the detection of diverse molecular species in living systems.

Intracellular pH has been mapped using thiol‐terminated aromatic acids, such as *p*‐mercaptobenzoic acid immobilized on AuNPs, whose carboxylate vibrational bands change with protonation and allows spatially resolved pH imaging in live cells [283, 284]. Reaction‐based SERS probes have also been developed for reactive sulfur species such as H_2_S [274, 285, 286], where reporter molecules on AuNPs undergo characteristic spectral changes upon reaction with H_2_S. For example, AuNPs functionalized with 4‐nitrothiophenol (4‐NTP) exhibit a characteristic peak at 1329 cm^−1^. In the presence of H_2_S, 4‐NTP is reduced to 4‐aminothiophenol (4‐ATP) and subsequently undergoes plasmon‐driven dimerization to *p*,*p*′‐dimercaptoazobenzene (DMAB), leading to a Raman scattering peak at 1139 cm^−1^. The nitrile stretching vibration of 4‐mercaptobenzonitrile (MPBN) at 2223 cm^−1^ serves as an internal standard and enables quantitative, ratiometric imaging of H_2_S (*I*_1139_/*I*_2223_ and *I*_1329_/*I*_2223_) (Figure 19c) [274]. Using these nanoprobes, ratiometric imaging of both exogenous and endogenous cellular H_2_S with submicromolar sensitivity was achieved (Figure 19d). More recent designs use reference Raman signals and membrane‐targeting ligands to quantify H_2_S release at the cell membrane with a 37 nM detection limit [287]. Besides H_2_S, Sun et al. developed a ratiometric SERS nanoprobe that selectively reacts with H_2_O_2_ for quantitative imaging of oxidative stress during ferroptosis in cancer cells [288].

In addition to small molecules and metabolites, SERS have been extended to peptides and nucleic acids [289]. Aβ peptides can nucleate gold nanoparticle assemblies to allow ratiometric SERS tracking of Aβ_1‐40_ aggregation from monomers to fibrils in neurons and brain tissue with picomolar sensitivity [290]. By combining nucleic acid recognition elements and Raman reporters on AuNPs, Zheng et al. developed miR‐21‐activated core‐satellite nanoprobes that undergo target‐induced assembly to generate ratiometric SERS signals for in vivo imaging of tumor‐associated miR‐21, with a detection limit of 0.16 pM [291].

## 8 Conclusions

The convergence of advanced materials and optical techniques is rapidly transforming how we interrogate cellular signals. A central theme is the growing shift toward label‐free and minimally perturbative optical detection. Materials‐based optical approaches have the potential to reduce phototoxicity, eliminate the need for genetic or chemical labeling, and support long‐term, high‐fidelity monitoring of cellular signals. The diverse material interfaces highlighted in this review are summarized in Table 1.

**TABLE 1:** Summary of materials‐based optical detection methods discussed.

Detection mechanism	Detection method	Biological specimen	Year	Detection SNR	Limit of detection	Measurement temporal resolution	Measurement spatial resolution
*Bioelectric signal*							
Polymer electrochromism	ECORE	HiPSC‐cardiomyocytes, chicken heart, rat heart, hippocampal neurons, dorsal root ganglion neurons, and brain slices	2020 [61, 62, 63, 65]	>200	∼3 µV	10 kHz	∼25 µm
Optically detected magnetic resonance shift in NV diamonds	Electron spin resonance magnetometry	*Myxicola infundibulum* worm, *Loligo pealeii* squid axons	2016 [210]	1.2 ± 0.1	∼80 mV	31 kHz	µm‐scale
Field‐sensitive optical transitions in graphene	CAGE	Chicken heart tissue	2021 [157]	—	∼15–20 µV	10 kHz	10–15 µm
Exciton‐trion conversion in MoS_2_	PL microscopy	HiPSC cardiomyocytes	2025 [193]	—	∼39–59 µV Hz^−1/2^	50 kHz	5 µm^2^
Birefringence‐driven polarization rotation in LCs	Optrode	Rabbit sinoatrial node and sciatic nerve	2022 [143]	−7.2 ± 16.1/6.6 ± 1.3 dB	10 µV	11 104 ± 672/620 ± 116 Hz	µm‐scale
Plasmonic and electrochemical impedance	P‐EIM	Rat hippocampal neurons	2024 [242]	—	—	100 µs	Subcellular
Localized surface plasmonic resonance	Electro‐plasmonic nanoantennas	HiPSC‐derived cardiomyocytes	2019 [258]	60–220	—	kHz	Multicellular/network level
*Biochemical signal*							
Polymer NP FRET and CRET	Fluorescence and chemiluminescence imaging	Murine macrophages, mice liver	2014 [76]	∼4	∼5–10 nM	—	Organ level
Polymer NP FRET	Fluorescence and afterglow luminescence imaging	Human blood, liver tumor and live mice	2020 [83]	71.9 ± 8.8	∼0.1 µM	—	∼3 mm
FRET‐based fluorescence quenching/recovery in GO	Fluorescence microscopy	MCF‐7 cells	2014 [162]	—	∼10 µM	—	Single cell
		Human T lymphoblasts	2018 [173]	—	10 cells mL^−^^1^	—	Single cell
CoPhMoRe using functionalized SWCNTs	NIR imaging	*E. coli*, HEK293 cells, yeast cells	2017 [178]	—	∼3.5 nM	2 Hz	2–10 µm
		Acute mice striatal brain slices	2019 [179]	—	∼10 nM	0.4 ± 0.18 s	µm‐scale
Fluorescence quenching via energy transfer in MXene	Confocal microscopy	MCF‐7, HepG2 and LO2 cells	2019 [203]	3	0.8–6 nM	—	Subcellular
	Fluorescence microscopy	MCF‐7, HepG2, HeLa and MCF‐10A cells	2025 [204]	3	3–48 µM	—	Singe cell
Optically detected magnetic resonance shift in NV diamonds	Confocal microscopy with microwave irradiation	*Caenorhabditis elegans*	2020 [211]	—	1.4°C Hz^−1/2^	0.5–1 s	∼12 mm
	Confocal microscopy‐based magnetometry	J774A.1 macrophage	2021 [212]	—	∼nM	120 s	Subcellular
Polymer electrochromism	Visual inspection	*E. coli*	2019 [68]	—	10^1^–10^2^ CFU mL^−1^	—	—
	UV–vis‐NIR spectrophotometry	*E. coli* and *Staphylococcus aureus*	2020 [67]	—	10^4^ cells mL^−1^	—	—
Localized surface plasmonic resonance	Extraordinary optical transmission (EOT) microscopy	Hybridoma cells, K562 leukemia cells, human peripheral blood mononuclear cells, and mouse EL‐4 lymphoma cells	2023 [270]	—	—	<1 min	Single cell
Ratiometric SERS	Raman confocal microscopy	HeGp2 and A549 cells	2022 [274]	—	0.24 µM	—	Subcellular
*Biomechanical signal*							
Bead displacement in 2D	Classical TFM	Swiss 3T3 cells	1999 [120]	—	104 pN m^−1^	—	∼5 µm
	STED‐TFM	RBL cells	2016 [127, 129]	—	—	0.05 fps (FOV:10 µm^2^)	∼500 nm
	FBSR TFM	U2OS, U‐251 glioma, and DCIS.COM lifeact‐RFP cells	2020 [126]	∼7	—	0.05 fps (FOV: 127 µm^2^)	—
Bead displacement in 3D	Confocal‐based multidimensional TFM	Mouse embryonic fibroblast	2012 [125]	—	300 Pa	—	∼10 µm
	3D‐SIM‐TFM	HeLa cells	2019 [128]	—	—	0.1 z‐stack fps (FOV: 30 µm^2^ (*xy*), 2 µm (*xz*)	∼2 µm
Diffraction intensity change	PDMS diffraction grafting	Rat cardiac cells	2017 [130]	—	—	32 fps	—
Vertical post deflection	PDMS microneedle array	Bovine pulmonary artery smooth muscle cells and NIH/3T3 mouse fibroblasts	2003 [131]	—	12 nN	—	9 µm
Optical cavity with elastomer	ERISM/WARP	NIH‐3T3 fibroblasts, primary human macrophages, Neonatal murine cardiomyocytes	2021 [229]	—	∼2 nm	100 fps	µm‐scale
	WARP	*Drosophila* larvae	2024 [230]	—	—	—	µm‐scale
Optical cavity with fluorescent dye	Interferometric biosensor	HiPSC‐derived cardiomyocytes	2024 [231]	∼10	—	—	30 µm
Plasmonic	SPR	Neonatal rat cardiomyocytes	2019 [15]	—	—	—	Multicellular
Plasmonic scattering	PSM	MCF‐7 cells	2025 [244]	—	—	200 fps	<1 µm lateral
Plasmonic scattering of AuNRs	LSPR and single particle tracking	A549 cells	2017 [260]	—	—	—	Subcellular

The materials selection depends on the cellular signal being measured. Conjugated polymers are particularly attractive for detecting fast bioelectric and biochemical signals because their optical properties can respond directly to changes in local potential or ionic environment. However, long‐term measurement stability and changes in optical or electrical properties during prolonged exposure to biological environments remain important challenges. This requires further chemical and structural optimization. For polymer nanoparticle energy‐transfer sensors, future development should also emphasize reversible and continuous measurements, as well as quantitative characterization of response kinetics, which is essential for monitoring sustained biochemical dynamics.

Polymer gels or elastomers are suitable for measuring cellular forces and interactions with the ECMs. However, techniques such as TFM remain limited by imaging hardware, particularly acquisition frame rates. In addition, although super‐resolution methods can improve optical imaging resolution, the effective spatial solution for resolving traction forces may still be limited by factors such as marker density and force‐reconstruction algorithms (Table 1) [127, 128, 129]. This motivates the development of material interfaces and optical readouts that enable faster force mapping.

Carbon‐based materials, 2D materials and quantum materials enable the detection of biochemical species, temperature, magnetic fields, and metabolic activities. However, broader adoption of these material platforms will require improved biocompatibility, reduced optical heating and simpler instrumentation. Finally, plasmonic material platforms are well‐suited for monitoring molecular binding, membrane deformations, and intracellular transport because they provide strong optical signals and rapid responses to local refractive‐index changes. Gold is the most widely used material for SPR and LSPR sensing because it is stable, biocompatible and easy to functionalize. Silver is often preferred for SERS because it produces stronger local electromagnetic enhancement, although it is less stable in biological environments. Aluminum and copper offer useful optical properties in selected spectral ranges, but their broader use is limited by oxidation [292, 293]. Future efforts should focus on improved surface chemistry and scalable fabrication methods that preserve optical performance while increasing stability and reproducibility in biological environments. Across these platforms, optical signals often arise from overlapping electrical, chemical, and mechanical processes at the biointerfaces, which can complicate data interpretation. Establishing quantitative frameworks that directly link optical observables to the underlying biological mechanisms will be critical.

Moving beyond passive optical detection, multimodal integration of platforms that simultaneously sense and modulate cellular electrical, chemical, and mechanical signals will provide a more holistic understanding of cell functions. Nanostructures such as particles, rods, or wires composed of gold, silicon, or other plasmonic materials can convert optical energy into localized thermal or electrical perturbations, therefore modulating membrane capacitance and ion channel activity to enhance or inhibit neuronal firing without the need for genetic modification [294, 295]. Similarly, 2D materials such as graphene or MXene offer neuromodulation capabilities that can be integrated with bioelectric recording approaches to achieve simultaneous control and monitoring of neuroactivities [296, 297]. In the biomechanical signal domain, polymer gels and elastomers can not only serve as sensing substrates but also enable programmable modulation of the cell microenvironment, where tunable stiffness and viscoelasticity regulate cell morphology, migration, and differentiation [109, 298, 299, 300]. These examples highlight a broader transition from static measurement toward dynamic, bidirectional biointerfaces that both read and write biological signals.

The field will also be poised to benefit from several other emerging directions. First, synthetic biology and materials science are beginning to converge, enabling programmable and cell‐specific assembly of functional optical interfaces directly at the cell surface [301, 302]. This paradigm shift, from placing cells on sensors to enabling cells to construct their own sensing interfaces, opens new opportunities for spatial precision and biological integration. Second, advances in artificial intelligence (AI)‐guided materials design and high‐throughput screening may accelerate the discovery of materials with tailored optical responses optimized for both signal detection and modulation [303]. Ultimately, we foresee materials‐based optical biointerfaces evolving from tools for observation into platforms for interactive and adaptive control of living systems. Continued progress in this field will deepen our understanding of fundamental biology and lay the groundwork for next‐generation diagnostic, therapeutic, and biohybrid systems that seamlessly bridge sensing and modulation. More broadly, clinical translation of these platforms beyond proof‐of‐concept studies will require reproducible device and sensor fabrication, and validation in complex biological samples across multiple laboratories and diverse patient populations.

## Author Contributions

X.R., J.R., Z.Z., and K.E.K. performed the literature review, organized the manuscript content, made the figures, and wrote the manuscript. F.L.H. contributed to making the figures. Y.P.Z. conceived the review article, developed the overall structure and scope, and wrote and edited the manuscript.

## Funding

This work was supported by the American Heart Association Career Development Award (https://doi.org/10.58275/AHA.25CDA1436867.pc.gr.229603), the Roy J. Carver Charitable Trust Individual Investigator Award (26‐6117), the University of Illinois Urbana‐Champaign Department of Materials Science and Engineering NEXUS seed grant, and Scialog grant #SA‐NCE‐2026‐045A from Research Corporation for Science Advancement and Allen Family Philanthropies.

## Conflicts of Interest

The authors declare no conflicts of interest.

## Data Availability

Data sharing is not applicable to this article as no new data was generated or analyzed. All information discussed in this review is derived from previously published studies cited in the article.
